# The sperm survival switch: circRNA-mediated regulatory loops control apoptotic signalling in human spermatozoa

**DOI:** 10.1038/s41420-026-03262-0

**Published:** 2026-07-25

**Authors:** Alice Luddi, Antonella Migliaccio, Matteo Prisinzano, Teresa Chioccarelli, Eugenia Annunzi, Francesca Paola Luongo, Francesca Girolamo, Maryam Raeispour, Bodo Levkau, Guillaume Martinez, Charles Coutton, Michele Caraglia, Caterina Bernacchioni, Gilda Cobellis, Chiara Donati, Francesco Manfrevola, Rosanna Chianese, Paola Piomboni

**Affiliations:** 1 Department of Molecular Medicine and Development, University of Siena, Siena, Italy; 2 Department of Experimental Medicine, University of Campania “Luigi Vanvitelli”, Naples, Italy; 3 Department of Experimental and Clinical Biomedical Sciences “Mario Serio”, University of Florence, Florence, Italy; 4 Departmental Faculty of Medicine and Surgery, Saint Camillus International University of Health Sciences, Rome, Italy; 5 Institute of Molecular Medicine III, Heinrich Heine University, Düsseldorf, Germany; 6 Hôpital Couple-Enfant, Centre Hospitalier Universitaire de Grenoble, UM de Génétique Chromosomique, Grenoble, France; 7 Genetic Epigenetic and Therapies of Infertility, Institute for Advanced Biosciences INSERM U1209, CNRS UMR5309, Grenoble, France; 8 Department of Precision Medicine, University of Campania “Luigi Vanvitelli”, Naples, Italy

**Keywords:** Apoptosis, Lipid signalling

## Abstract

Male reproductive health is declining worldwide, partly due to lifestyle factors including obesity, which is linked to increased sperm apoptosis and impaired assisted reproductive technologies (ARTs) outcomes. This growing issue underscores the urgent need for novel molecular biomarkers capable of improving sperm quality assessment. In this context, circular RNAs (circRNAs) are emerging as regulators of germ cell functions and apoptosis, but their role as early apoptotic markers in human sperm under lifestyle-related stress remains poorly explored. In this study, by using the obesity as experimental condition of sperm apoptosis-induction, we performed a comprehensive circRNA profiling in High-quality spermatozoa (A-SPZ) collected from Normal-weight and Obese men. Obese sperm exhibit a pro-apoptotic phenotype associated with a cargo of differentially expressed (DE-) circRNAs enriched in pro-apoptotic regulatory networks. Mechanistically, circRNAs arise both from the trigger of FUS-regulated endogenous backsplicing and *via* extracellular vesicles (EVs) mediated delivery, thereby establishing a functional pro-apoptotic circRNA regulatory circuit. Furthermore, Obese men exhibit a dysregulated sphingosine 1-phosphate (S1P) signaling axis, along with an imbalanced S1P–to–ceramide rheostat within EVs and increased levels of pro-apoptotic ceramide. Finally, the in vitro sperm apoptosis induction confirmed a functional circRNA–apoptosis axis, identifying a subset of circRNAs responsive to pro-apoptotic stimuli that engage in a self-reinforcing regulatory loop involving pro-CASPASE3 activation. Our findings elucidate a pro-apoptotic propensity in Obese A-SPZ positioning circRNAs as putative molecular biomarkers candidate for early apoptosis. These spermatic circRNAs, which are significantly up-regulated under apoptotic conditions, exert a dual functional role: (i) serving as diagnostic markers for early-stage apoptosis and (ii) acting as active molecular adapters to enhance apoptosis activation. These circRNAs could be suggested as early sperm apoptotic biomarkers enhancing the precision of sperm quality assessment and, in turn, the success of ART procedures.

## Introduction

Over the past few decades, the male reproductive health has undergone a marked decline, especially in young world-wide population, attracting growing scientific attention as a global health issue [1–6]. A definitive causal framework has yet to be established and several lines of evidence support a multifactorial etiology. Unhealthy lifestyle habits (i.e., tobacco smoking and alcohol consumption), metabolic dysfunctions (i.e., obesity condition) and the widespread exposure to environmental toxicants have all been associated with defective spermatogenesis and sperm quality decline [7, 8]. Indeed, all lifestyle stressors are associated with a wide spectrum of sperm anomalies, including: (i) reduced ejaculate volume, (ii) decreased sperm concentration and motility and (iii) higher incidence of morphological defects [9–16]. Intriguingly, although these risk factors differ in their origin and mechanistic pathways, ranging from metabolic imbalance and endocrine disruption to oxidative stress and genotoxicity, they ultimately converge on a common endpoint: the activation of sperm apoptosis. This programmed cell death is an alarming hallmark of male reproductive decline [17].

In this light, assisted reproductive technologies (ARTs) are increasingly employed to overcome fertility challenges as conventional semen parameters (sperm concentration, sperm motility, sperm morphology) frequently fail to accurately predict clinical outcomes in ART procedures [17, 18]. Notably, burgeoning evidence has highlighted a potential correlation between sperm apoptosis and suboptimal ART success rates [17, 19]. Within this framework, the sperm DNA fragmentation (SDF) index has emerged as a widely adopted sperm apoptotic marker, linked to lower fertilization rates and reduced pregnancy success [20, 21]. However, the SDF index appears to be indicative of late events in the apoptotic cascade, and thus unable to detect the early stage of sperm apoptosis [17]. Hence, the identification of newest sperm apoptotic markers could improve the prediction of ART success in clinical practices.

For this purpose, circular RNAs (circRNAs) appear promising markers suitable for monitoring cell apoptosis and sperm morpho-functional skills. At testis level, *circSMC1B* modulates bovine male germline stem cell apoptosis [22], while in boar spermatozoa (SPZ), *circCREBBP* promotes sperm motility and inhibits apoptosis through the sponging of miR-10384 and miR-143-3p [23]. In terms of sperm quality assessment, the circRNA profiling carried out in human SPZ collected from normozoospermic men and asthenozoospermic patients have highlighted their potential application as molecular markers related to sperm quality [24–26]. Accordingly, the responsiveness of sperm circRNA cargo to multiple lifestyle stressors has been also characterized [26, 27]. Despite this expanding body of evidence, the specific involvement of circRNAs in sperm apoptotic pathways, along with the molecular mechanisms underlying their putative pro-apoptotic action and their potential role as early spermatic apoptotic biomarkers for enhancing ART outcomes, remains largely unexplored.

In this scenario, bioactive sphingolipids, in addition to their functions in membrane structure, play a critical role in regulating cell fate. The well-known sphingosine-1-phosphate (S1P)–to–ceramide rheostat is one of the most important cellular mechanisms governing the balance between cell survival and apoptosis [28]. Specifically, among these two interconvertible bioactive lipids, S1P mainly promotes pro-survival processes while ceramide is mostly linked to programmed cell death. Furthermore, both molecules influence sperm biological functions by modulating reactive oxygen species (ROS) production and calcium signaling, respectively [29].

In the present study, leveraging human obesity as experimental paradigm of unhealthy lifestyle recognized for its deleterious impact on sperm survival, we assessed the apoptotic phenotype and performed an exhaustive circRNA microarray analysis in high-quality SPZ fraction (namely A-SPZ), collected from Normal-weight (Ctrl) and Obese men. Interestingly, several differentially expressed (DE-) circRNAs, bioinformatically predicted to modulate apoptotic signaling, were identified. These pro-apoptotic DE-circRNAs appear to be both transferred from seminal EVs and endogenously synthesized within sperm through Fused in Sarcoma (FUS) protein-dependent backsplicing. In vitro induction of sperm apoptosis induction corroborated a putative circRNA-apoptosis functional axis underling not only the pro-apoptotic phenotype characterized in Obese A-SPZ, but also revealing a subset of circRNAs responsive to pro-apoptotic stimuli which potentially participate in a self-reinforcing regulatory circuit governing the apoptotic fate of SPZ *via* pro-CASPASE3 activation. Furthermore, Obese men showed alterations in the S1P molecular machinery in both seminal plasma EVs and A-SPZ, along with an imbalanced S1P–to–ceramide rheostat within EVs, with an increase in the levels of pro-apoptotic specific ceramide species.

Taken together, these insights delineate an enhanced apoptotic propensity of high-quality sperm cells under obesity condition, underscoring the relevance of circRNAs as robust promising biosensors useful to refine sperm quality assessment and improve the success of ART procedures.

## Results

### Assessment of sperm apoptotic phenotype in Obese SPZ

**Fig. 1:**
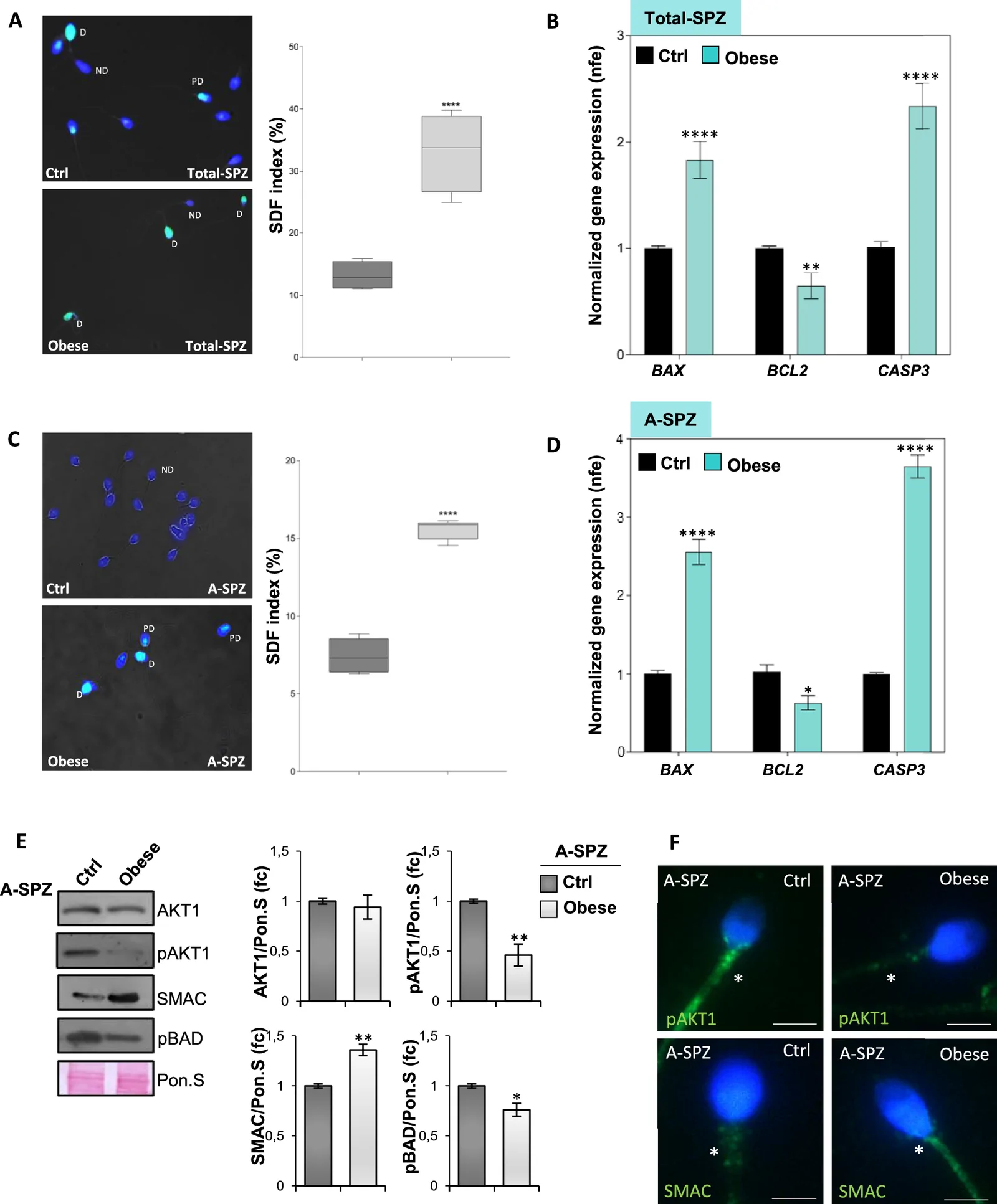
Assessment of apoptotic phenotype in Ctrl and Obese SPZ. **A**–**C** Immunofluorescence TUNEL assay in total SPZ (**A**) and A-SPZ (**C**) collected from Ctrl and Obese subjects. The representative fluorescence images showed different staining patterns: no DNA damage (ND), partial DNA damage (PD), complete DNA damage (**D**). Magnification 630X. The sperm DNA fragmentation index (SDF) was expressed as percentage and reported as mean ± SEM. **B**–**D** Expression analysis of *BAX, BCL2* and *CASP3* transcripts in total SPZ (**B**) and A-SPZ (**D**) collected from Ctrl and Obese subjects. RT-qPCR data were normalized using *GAPDH*, expressed as normalized fold expression (nfe) and reported as mean value ± S.E.M. **E** Western blot analysis of AKT1, pAKT1, SMAC and pBAD proteins in Obese compared with Ctrl A-SPZ. Signals were quantified by densitometry analysis and normalized to Ponceau Red (Pon.S). Data were expressed in OD values as fold change (fc) and reported as mean ± SEM. **F** Immunofluorescence analysis of pAKT (green) and SMAC (green) in Obese compared with Ctrl A-SPZ. Nuclei were labeled with DAPI (blue); white asterisks represent pAKT1 and SMAC localization in sperm tail. Scale bar: 4 μm. Statistical significance was assessed using the two-tailed Student’s *t* test or the non-parametric Mann–Whitney *U* test depending on data distribution normality (**A–D**: *n* = 15 biological replicates per group) or the Student’s *t* test (**E**: *n* = 8 biological replicates per group); *: *p* < 0.05, **: *p* < 0.01, ***: *p* < 0.001, ****: *p* < 0.0001.

Obesity was chosen as a robust experimental model to elucidate previously unexplored molecular mechanisms governing the activation of apoptotic signaling pathways in sperm cells. To this end, the total SPZ population was collected from obese (Obese experimental group) and normal weight (Ctrl experimental group) subjects. To substantiate the obesity-mediated sperm apoptotic phenotype, we performed a TUNEL immunofluorescence assay in Ctrl- and Obese-SPZ, subsequently determining the relative SDF index. Compared with the Ctrl group, the analysis revealed a high incidence of SPZ exhibiting TUNEL-positive fluorescence signal, characterized by a partial (PD) or complete (D) DNA damage, in Obese men (Fig. 1A). Accordingly, a significant increase (*p* < 0.0001) of spermatic SDF index occurred in Obese-SPZ (Fig. 1A). The expression analysis of anti- (*BCL2*) and pro- (*BAX; CASP3*) apoptotic genes was assessed in Ctrl- and Obese-SPZ, by RT-qPCR analysis. As showed in Fig. 1B, a significant (*p* < 0.01) reduction of *BCL2* accomplished to a significant increase (*p* < 0.0001) of *BAX* and *CASP3* transcripts was detected in Obese vs Ctrl-SPZ, confirming the validity of the experimental model.

Considering that sperm cells represent a heterogeneous population comprising both high- (A-SPZ) and low- (B-SPZ) quality SPZ, which differ in morpho-functional parameters [23], we conducted a deeper investigation of the apoptotic phenotype just focusing on A-SPZ collected from Ctrl and Obese men by density gradient centrifugation. To ensure the robustness of methodology, we first validated the sperm isolation technique to confirm the efficient enrichment of a healthy sperm population; this was achieved through Annexin V-based immunofluorescence analysis performed on both Total- and the A-SPZ fractions. As reported in Supplementary Fig. 1, compared to Total-SPZ, a significant reduction (*p* < 0.001) in the percentage of Annexin V-positive cells, representing SPZ with an early apoptotic phenotype, was observed in the A-SPZ population. This finding demonstrated that the sperm isolation procedure effectively enhances the enrichment of high-quality SPZ. Therefore, the persistence of an early apoptotic phenotype following the density gradient centrifugation protocol may be attributed to the intrinsic physiological status of the sperm cells rather than to the isolation procedure itself.

The TUNEL assay analysis showed a higher incidence of positive fluorescent signal, associated with a significant (*p* < 0.0001) increase of spermatic SDF index, in Obese than Ctrl A-SPZ (Fig. 1C). Coherently, the RT-qPCR analysis revealed a significant (*p* < 0.05) reduction of *BCL2* anti-apoptotic marker, associated with significantly increased levels (*p* < 0.0001) of both *BAX* and *CASP3* transcripts in Obese compared with Ctrl A-SPZ (Fig. 1D).

To further confirm the reasonable obesity-mediated early apoptosis in A-SPZ fraction, we determined by western blot analysis the cellular content of AKT1, phosphorylated-AKT (pAKT1), SMAC (DIABLO) and phosphorylated-BAD (pBAD) proteins, here chosen as critical modulators of sperm apoptosis [30, 31], in A-SPZ collected from Ctrl and Obese men. The obtained results showed that AKT1 protein content was unchanged in both experimental groups, whereas a significant reduction (*p* < 0.01) of the relative phosphorylated form, pAKT1, occurred in Obese compared with Ctrl A-SPZ (Fig. 1E). Relatively to Ctrl group, SMAC and pBAD protein levels were significantly (*p* < 0.01; *p* < 0.05) higher and lower in Obese A-SPZ, respectively (Fig. 1E). In accordance with previous findings [30, 31], the immunofluorescence analysis revealed an intense pAKT1 and SMAC signals specifically detectable in the sperm tail, at the level of the midpiece region (Fig. 1F). However, a less intense pAKT1 fluorescent signal was detected in Obese compared with Ctrl A-SPZ (Fig. 1F). Conversely, a higher SMAC fluorescent signal occurred in Obese than Ctrl A-SPZ (Fig. 1F), thus validating Western blot data and demonstrating that the obesity condition shifts the balance between anti- and pro-apoptotic factors in favor of the latter.

### Identification of DE-circRNAs in Obese compared to Normal-weight derived A-SPZ fraction

Sperm cells represent a heterogeneous population comprising both high- (A-SPZ) and low- (B-SPZ) quality cells, diverging in terms of morpho-functional parameters [23]. To elucidate the potential role of circRNAs in modulating the apoptotic pathway in sperm cells, and their application as early spermatic apoptotic biomarkers, sperm circRNA cargo was profiled in A-SPZ collected from Normal-weight (Ctrl) and Obese men by using a microarray experimental strategy.

**Fig. 2:**
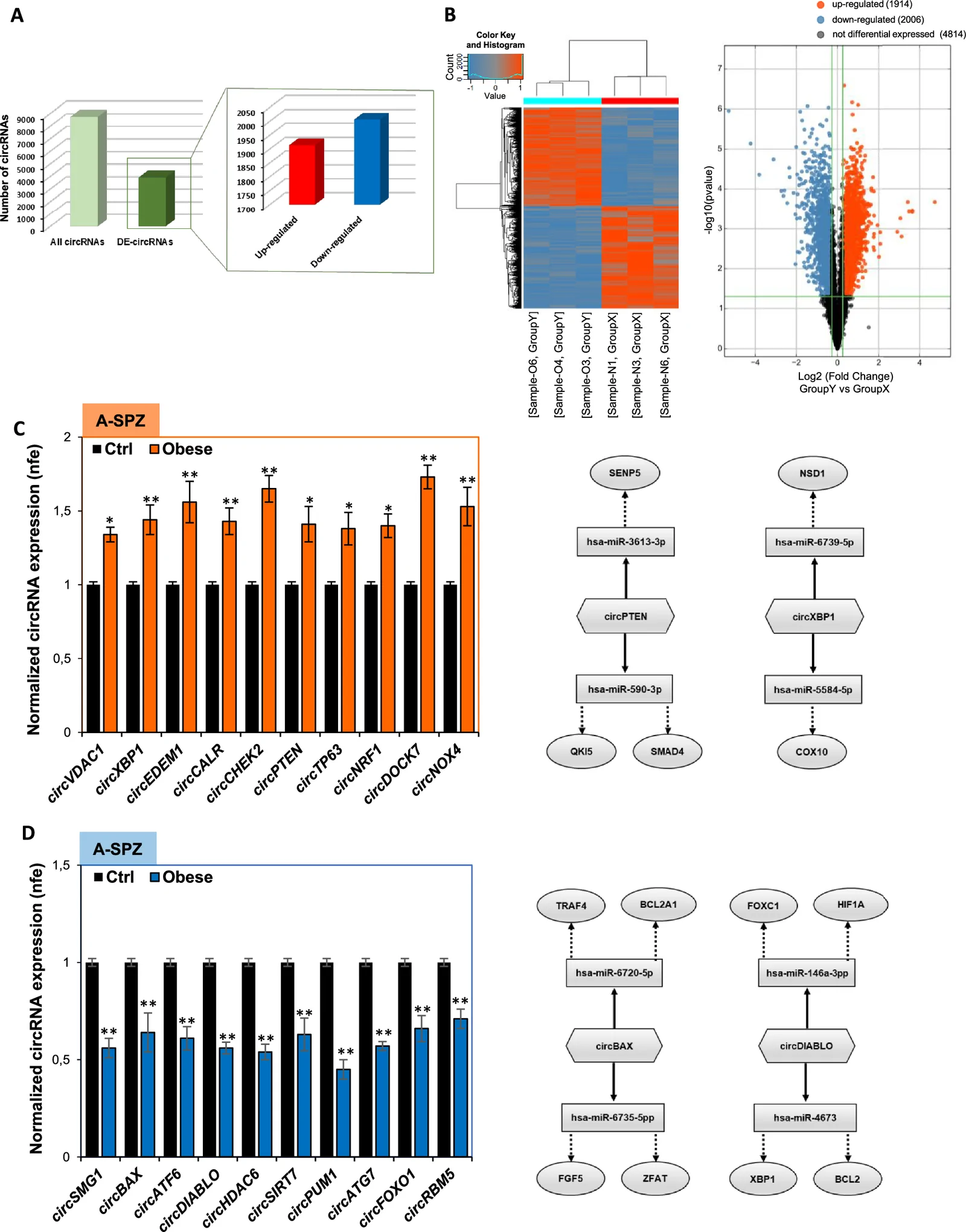
Differential expression of circRNAs between Ctrl and Obese A-SPZ. **A** The distribution of 3920 DE-circRNAs (1914 up- and 2006 down-regulated), among a total number of 8734 circRNAs identified in Obese compared with Ctrl A-SPZ. **B** Hierarchical clustering analysis of DE-circRNAs in Ctrl (samples N-1, N-3, N-6) and Obese (samples O-3, O-4, O-6) A-SPZ; the expression values were represented in different colors, indicating expression levels above and below the median expression level across all samples. The volcano plot was constructed using fold change and *p*-values; in detail, the values on X and Y axes are log2 (FC = fold change) and –log10 (*p*-values), respectively. **C** Expression analysis of 10 circRNAs up-regulated in Obese compared with Ctrl A-SPZ. RT-qPCR data were normalized using *GAPDH* expressed as fold expression (nfe) and reported as mean value ± S.E.M. CeRNET of 2 circRNAs up-regulated in Obese A-SPZ involved in apoptotic pathways. Networks were built using Cytoscape. Hexagonal and rectangular symbols represent circRNAs and miRNAs, respectively. The arrow indicates the tethering activity of circRNAs. **D** Expression analysis of 10 circRNAs down-regulated in Obese compared with Ctrl A-SPZ. RT-qPCR data were normalized using *GAPDH* expressed as fold expression (nfe) and reported as mean value ± S.E.M. CeRNET of 2 circRNAs down-regulated in Obese A-SPZ involved in apoptotic pathways. Networks were built using Cytoscape. Hexagonal and rectangular symbols represent circRNAs and miRNAs, respectively. The arrow indicates the tethering activity of circRNA. Statistical differences were evaluated using the two-tailed Student’s *t* test (**C**, **D**: *n* = 8 biological replicates per group); *: *p* < 0.05; **: *p* < 0.01.

A total number of 8734 circRNAs was identified in A-SPZ fraction (Fig. 2A). The identified circRNAs were finely analyzed using stringent statistical parameters (fold change cut-off >1.2 and *p*-values cut-off <0.05) to recognize the significative DE-circRNAs in Obese A-SPZ. Relatively to Ctrl group, a number of 3920 DE-circRNAs, clustered in 1914 up- and 2006 down-regulated, was determined in Obese A-SPZ (Fig. 2A). Heatmap and volcano plot analyses were employed to represent DE-circRNAs, clustered based on their fold change and variation in expression levels between Obese and Ctrl A-SPZ experimental groups (Fig. 2B). According to their structural parameters, the profiled DE-circRNAs were clustered to identify the predominant circRNA biotypes in A-SPZ. As reported, exonic circRNAs represented the most abundant type of spermatic DE-circRNA (84%) identified in Obese A-SPZ, followed by intronic (7%) and sense-overlapping (6%). Interestingly, antisense and intergenic (2% and 1%, respectively) appeared to be less represented (Supplementary Fig. 2A). Depending on the genome location of their host genes, DE-circRNAs were widely distributed across all chromosomes. While a comparable number of DE-circRNAs was uniformly produced by most chromosomes, interestingly the chromosomes 13, 18, 21 and 22 produced the lowest number of DE-circRNAs. In addition, a low number of DE-circRNAs was generated from X and Y chromosomes as well as from the mitochondrial (M) chromosome (Supplementary Fig. 2B). In order to define the biological and molecular pathways potentially modulated by the DE-circRNAs profiled in Obese A-SPZ, the GO functional enrichment and KEGG pathway bioinformatic analyses were performed (Supplementary Figs. 3 and 4, respectively). Relatively to GO top scores, both up- and down-regulated DE-circRNAs in Obese A-SPZ were involved in metabolic biological processes including protein metabolic process, primary metabolic process and cellular response to stress (Supplementary Fig. 3A, B). Conversely, KEGG pathway analysis showed the conceivable DE-circRNA participation in molecular processes associated to cell apoptosis, such as cellular senescence and ubiquitin mediated proteolysis pathways (Supplementary Fig. 4A, B).

To assess the putative functional involvement of DE-circRNAs in the regulation of sperm apoptosis, a panel of 20 DE-circRNAs (10 up- and 10 down-regulated in Obese A-SPZ), predicted to participate in apoptotic pathways, was selected for the validation of circRNA microarray results using RT-qPCR analysis (Fig. 2C, D). RT-qPCR data revealed a significant increase (*p* < 0.01; *p* < 0.05) in the expression levels of all predicted up-regulated circRNAs (*circVDAC1, circXBP1, circEDEM1, circCALR, circCHEK2, circPTEN, circTP63, circNRF1, circDOCK7*, and *circNOX4*) in Obese compared with Ctrl A-SPZ (Fig. 2C). To further strengthen the functional correlation between circRNAs and the modulation of apoptotic pathways in sperm cells, we performed a bioinformatic construction of molecular ceRNETs for circPTEN and circXBP1, as their linear counterparts encode for proteins known to regulate apoptosis either directly—as master regulators—or indirectly, through modulation of endoplasmic reticulum (ER) stress, mitochondrial dysfunction, and the unfolded protein response (UPR) [32–36]. In line with these predictions, the top miRNAs sponged by the selected circRNAs (hsa-miR-590-3p, hsa-miR-3613-3p, hsa-miR-5584-5p, and hsa-miR-6739-5p) were found to modulate downstream mRNA targets previously described as molecular regulators of apoptotic pathways as: (i) QKI5 modulates spermatogonia and spermatocyte apoptosis by binding specific long non-coding RNAs [36, 37], (ii) *SMAD4* overexpression is associated with germ cell apoptosis and spermatogenic arrest [38], (iii) *SENP5* overexpression promotes apoptosis in murine cardiomyocytes [39, 40], (iv) *COX10* dysfunctions enhances apoptotic pathway activation [41] and (v) *NSD1* mutations correlate with a high incidence of apoptosis in embryos [42] (Fig. 2C).

Consistently with microarray data, the expression levels of the predicted down-regulated circRNAs (*circSMG1, circBAX, circATAF6, circDIABLO, circHDAC6, circSIRT7, circPUM1, circATG7, circFOXO1*, and circRBM5) were significantly reduced (*p* < 0.01) in Obese A-SPZ (Fig. 2D), further confirming the validity of microarray analysis. The molecular ceRNETs built for *circBAX* and *circDIABLO*, whose linear counterparts encode for well-known proteins that actively modulate apoptosis onset [43], revealed several top sponged miRNAs (hsa-miR-6735-5p, hsa-miR-6720-5p, hsa-miR-146a-3p, and hsa-miR-4673) potentially regulating downstream mRNA targets encoding for apoptosis-related factors, as: (i) FGF5 exerts anti-apoptotic effects through activation of the PI3K/AKT signaling pathway [44], (ii) ZFAT acts as a critical regulator of apoptosis and cell survival [44, 45] and (iii) TRAF4, BCL2A1, XBP1, HIF1A, and BCL2 function as anti-apoptotic factors [46–49], and (iv) FOXC1 contributes to cell survival mechanisms [50].

### Assessment of EV circRNA cargo, sphingolipid profile and S1P signaling pathway

Consistent with the pro-apoptotic functional profile of up-regulated circRNAs predicted *via* ceRNET bioinformatic analysis, this study investigated the regulatory mechanisms driving their increase in Obese A-SPZ, with the aim to elucidate the circRNA-mediated molecular pathways underlying the onset of sperm apoptosis. To this end, complementary experimental strategies were employed. First, the morpho-molecular characteristics of EVs isolated from Ctrl and Obese seminal plasma were defined. Next, their potential role in modulating the up-regulated sperm circRNAs was evaluated, aiming to assess the specific contribution of epididymosomal and/or prostasomal sources.

**Fig. 3:**
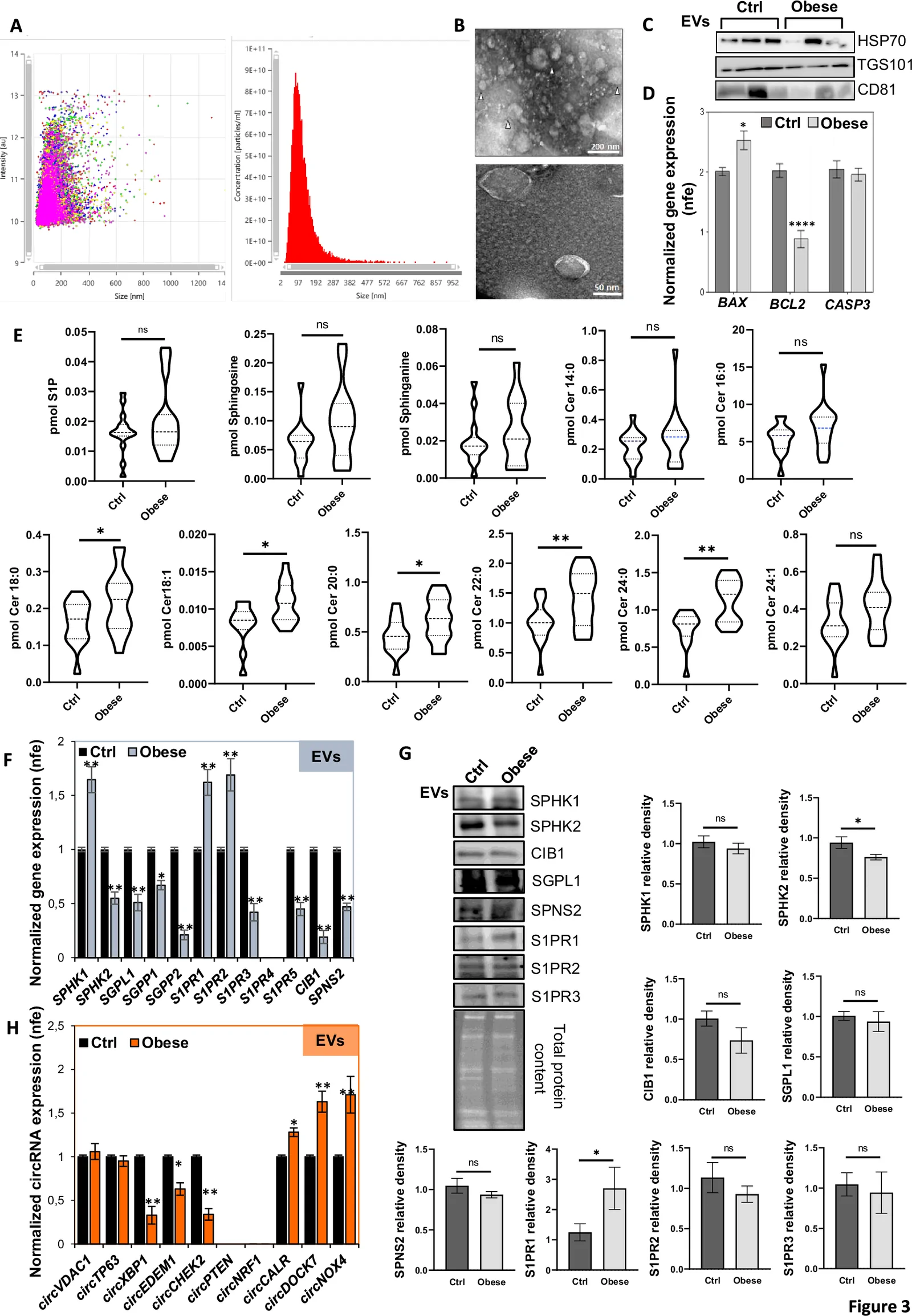
Differential expression of circRNAs between Ctrl and Obese A-SPZ. **A** Averaged particle size distributions (left panel; each colored symbol represents an independent technical replicate) and concentration (right panel) for seminal EVs collected from Ctrl and Obese seminal plasma. **B** Representative electron-micrographs of EVs/Exosomes collected from seminal plasma, visualized after negative staining. **C** Western blot analysis of EV molecular markers (HSP70, TSG101, CD81). Data showed a representative image of 3 independent experiments. **D** Expression analysis of *BAX, BCL2* and *CASP3* transcripts in seminal EVs collected from Ctrl and Obese seminal plasma. RT-qPCR data were expressed as normalized fold expression (nfe) and reported as mean value ± S.E.M. **E** LC-MS/MS sphingolipidomic analysis in EVs collected from Ctrl and Obese seminal plasma. The levels of S1P, Sphingosine, Sphinganine and Ceramides (Cer 14:0, Cer 16:0, Cer 18:0, Cer 18:1, Cer 20:0, Cer 22:0, Cer 24:0 and Cer 24:1) were normalized on the original seminal liquid volume from which EVs were isolated. **F** Expression analysis of lipidomic markers (*SPHK1, SPHK2, SGPL1, SGPP1, SGPP2, S1PR1, S1PR2, S1PR3, S1PR4, S1PR5, CIB1, SPNS2*) in seminal EVs collected from Ctrl and Obese subjects. RT-qPCR data were normalized using *GAPDH* expressed as fold expression (nfe) and reported as mean value ± S.E.M. **G** Western blot analysis of SPHK1, SPHK2, CIB1, SPL, SPNS2, S1PR1, S1PR2 and S1PR3 in protein lysates of Ctrl or Obese seminal fluid-derived EVs. Signals were quantified by densitometry analysis and normalized to the total protein content considering the intensity of all proteins in the lane. Data were expressed in relative density. **H** Expression analysis of 10 up-regulated circRNAs in seminal EVs collected from Ctrl and Obese seminal plasma. RT-qPCR data were normalized using *GAPDH* expressed as normalized fold expression (nfe) and reported as mean value ± S.E.M. Statistical differences were computed using the independent two-tailed Student’s *t* test or Mann–Whitney *U* test depending on data distribution normality (**D**–**H**: from *n* = 10 up to *n* = 14 biological replicates per group); *: *p* < 0.05; **: *p* < 0.01; ****: *p* < 0.0001.

The EVs isolated from Ctrl and Obese seminal plasma were characterized by NTA for their EV content and size (Fig. 3A). The amount of nanosized particles was similar between Ctrl and Obese samples (Fig. 3A). Although the size of particles peaked around 100 nm diameter in both experimental groups, a broad size distribution was observed. Comparable particle concentration ranging from 1 × 10^10^ to 9 × 10^10^/mL, and homologous size distribution, revolving around the predicted 100 nm peak, was detected in all samples (Fig. 3A). This structural identity of isolated EVs was confirmed by TEM analysis, evidencing the typical EV size and morphology across the Ctrl and Obese samples (Fig. 3B). We evidenced a vesicle-feature-characteristic round-shaped structure and a thin electron dense membrane. EVs were heterogeneous in size, with diameters ranging from 50 to 150 nm (Fig. 3B). In addition, molecular characterization of EVs performed by Western blot analysis, confirmed that all samples express exosomal markers HSP70, TGS101 and CD81, consistent with the presence of small EVs/exosomes (Fig. 3C). As showed in Fig. 3D, a significant increase (*p* < 0.05) of *BAX* accomplished to a significant reduction of *BCL2* (*p* < 0.0001) transcript was detected in Obese *vs* Ctrl-derived EVs.

In view of the critical role exerted by sphingolipids in the control of cell viability and apoptosis [28], an LC-MS/MS analysis was conducted in EVs isolated from Ctrl and Obese seminal plasma to investigate putative differences in bioactive sphingolipid enrichment. As shown in Fig. 3E, Cer 18:0, Cer 18:1, Cer 20:0 (*p* < 0.05), Cer 22:0 and Cer 24:0 (*p* < 0.01) levels were significantly increased in the Obese experimental group. Conversely, no changes were detected for Cer 14:0, Cer 16:0, Cer 24:1, S1P, sphingosine and sphinganine levels in Obese EVs. These results highlighted a dysregulation of lipid species embedded in EVs derived from seminal plasma of Obese men, suggesting that increased EV ceramide levels could potentially act as pro-apoptotic stimulus in the recipient sperm cells (Fig. 3E).

Since S1P signaling axis has been demonstrated to play a role in exosomal cargo sorting [51], we then analyzed the expression of S1P signaling key components in EVs both at mRNA and protein level in order to assess whether this pathway could contribute to the modulation of circRNA sorting within EVs and to the EV-mediated delivery to SPZ. Specifically, the mRNA levels of genes involved in: (i) S1P synthesis (sphingosine kinase *SPHK1* and *SPHK2)*, (ii) S1P reversible and irreversible degradation (S1P phosphatases *SGPP1, SGPP2* and S1P lyase *SGPL1)*, (iii) SPHK plasma membrane translocation (Calcium- and Integrin-Binding Protein 1, *CIB1*), (iv) S1P specific transport (Spinster homolog 2, *SPNS2)*, and (v) S1P specific G-protein coupled receptors *(S1PR1, S1PR2, S1PR3, S1PR4, S1PR5*) were analyzed in EVs collected from Ctrl and Obese subjects, using RT-qPCR analysis (Fig. 3F). Relatively to the Ctrl group, RT-qPCR data revealed a significant increase (*p* < 0.01) of *SPHK1, S1PR1* and *S1PR2* levels in Obese compared with Ctrl EVs. Conversely, a significant (*p* < 0.01; *p* < 0.05) reduction of *SPHK2, SGPL1, SGPP1, SGPP2, S1PR3, S1PR5, CIB1* and *SPNS2* transcripts was observed in Obese EVs, whereas *S1PR4* transcript was not detected by RT-qPCR analysis (Fig. 3F). In parallel, the protein content of molecules involved in S1P metabolism and signaling was investigated at protein level by Western blot analysis in EVs collected from Ctrl and Obese subjects. Interestingly, all the analyzed proteins were found to be present in EVs, supporting the crucial role of the bioactive sphingolipid signaling in this biological context. A significant reduction (*p* < 0.05) of SPHK2 levels occurred in Obese EVs, while a significant increase (*p* < 0.05) of S1PR1 was detected in EVs derived from Obese patients in respect to Ctrl (Fig. 3G). Altogether, these results disclose alterations in the S1P molecular machinery at both transcriptional and protein levels in seminal plasma EVs of Obese men, with critical effects on SPZ homeostasis and functionality.

To confirm the plausible impaired circRNA sorting within EVs, the expression levels of the identified up-regulated circRNAs were investigated in EVs collected from Ctrl and Obese seminal plasma using RT-qPCR analysis. The qPCR data revealed distinct circRNA expression patterns in EVs derived from Obese subjects (Fig. 3H). Compared with Ctrl group, the expression levels of circRNAs previously validated as up-regulated in Obese A-SPZ were found to be unchanged (*circVDAC1, circTP63*), significantly reduced (*p* < 0.01; *p* < 0.05) (*circXBP1, circEDEM1, circCHEK2*), undetectable (circPTEN, circNRF1) or significantly increased (*p* < 0.01; *p* < 0.05) (*circCALR, circDOCK7, circNOX4*) in EVs derived from Obese seminal plasma (Fig. 3H), suggesting a potential EV-mediated delivery of certain circRNAs to sperm cells. To validate this hypothesis, we carried out an in vitro vesicle shuttle assay to demonstrate whether *circCALR*, *circDOCK7* and *circNOX4*, extremely enriched in obese-derived seminal plasma EVs, could be transferred to SPZ. In detail, the A-SPZ fraction collected from Ctrl subjects was in vitro incubated with (i) PBS as a vehicle control, (ii) Obese-derived seminal plasma (Obese-SP) or (iii) Obese-SP pretreated with the CD9 antibody, respectively. Following the treatment, the levels of *circCALR, circDOCK7* and *circNOX4* were analyzed by RT-qPCR analysis. As reported in Supplementary Fig. 5, a significant increase (*p* < 0.01) of all circRNAs investigated was observed when Ctrl A-SPZ were incubated with seminal plasma collected from Obese men. Interestingly, this enrichment was significantly attenuated by the anti-CD9 masking antibody, an experimental approach previously validated to successfully reduce the delivery of RNA molecules from EVs to SPZ [52] (Supplementary Fig. 5). Overall, the data suggested that some up-regulated circRNAs in Obese A-SPZ (*circCALR, circDOCK7, circNOX4*) could be supported by EV delivery, whereas other ones could be enriched *via* alternative pathways converging with the epididymal EV-mediated transfer.

### Obese A-SPZ display dysregulated S1P signaling axis and FUS protein levels

**Fig. 4:**
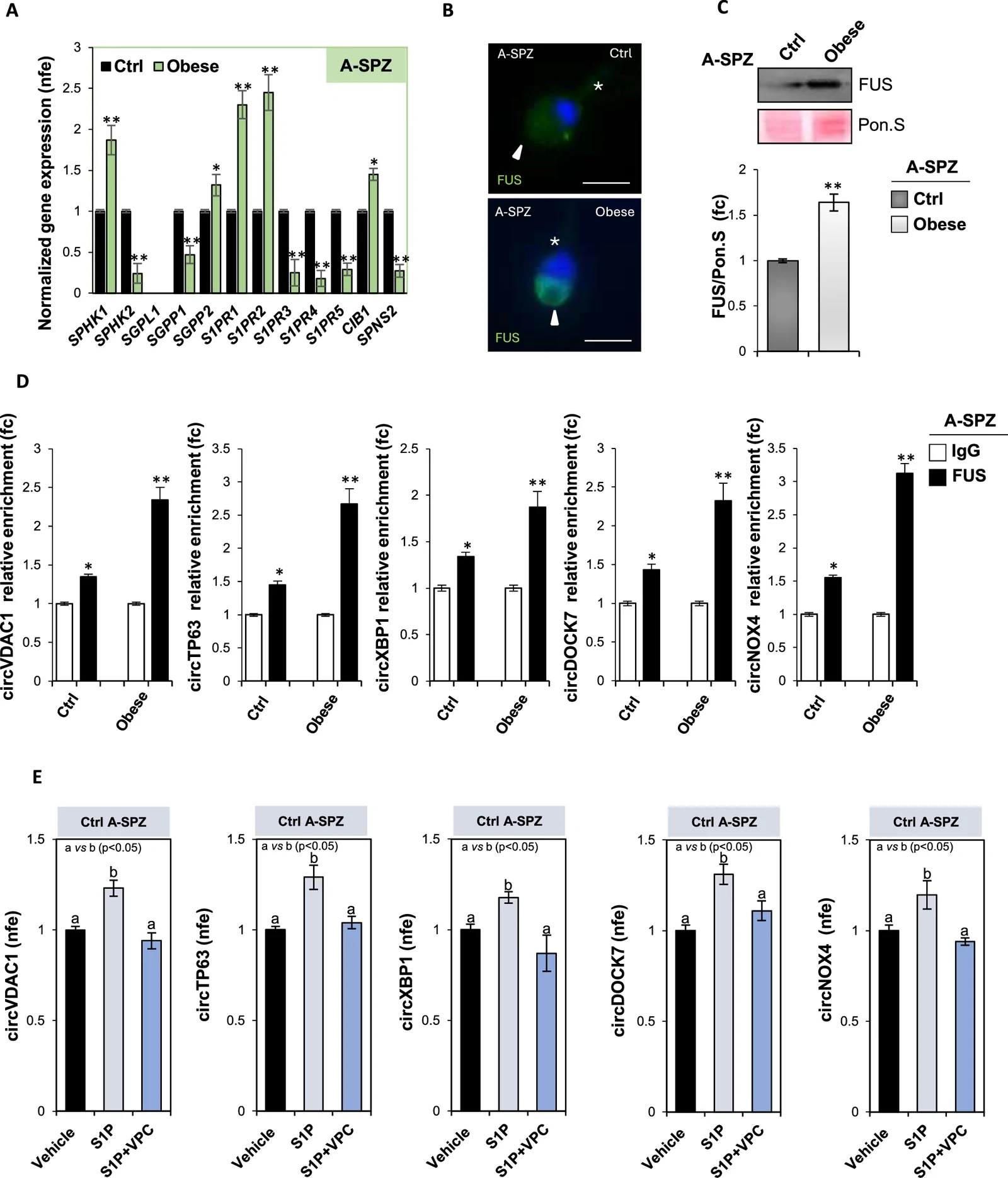
FUS protein orchestrates endogenous backsplicing in Obese A-SPZ. **A** Expression analysis of lipidomic markers (*SPHK1, SPHK2, SGPL1, SGPP1, SGPP2, S1PR1, S1PR2, S1PR3, S1PR4, S1PR5, CIB1, SPNS2*) in A-SPZ collected from Ctrl and Obese subjects. RT-qPCR data were normalized using *GAPDH* expressed as fold expression (nfe) and reported as mean value ± S.E.M. **B** Immunofluorescence analysis of FUS (green) in Obese compared with Ctrl A-SPZ. Nuclei were labeled with DAPI (blue); white arrowheads represent FUS localization in sperm head; white asterisks represent FUS localization in sperm tail. Scale bar: 4 μm. **C** Western blot analysis of FUS protein in Obese compared with Ctrl A-SPZ. Signals were quantified by densitometry analysis and normalized to Ponceau Red (Pon.S). Data were expressed in OD values as fold change (fc) and reported as mean ± SEM. **D** The enrichment levels of 5 circRNAs (*circVDAC1; circTP63; circXBP1; circDOCK7; circNOX4*) in the products of the RIP assay (FUS-IP compared with IgG-IP) in A-SPZ collected from Ctrl and Obese subjects, detected by RT-qPCR. Data were expressed in relative enrichment values as fold change (fc) and reported as mean ± SEM. **E** Expression analysis of 5 circRNAs (*circVDAC1; circTP63; circXBP1; circDOCK7; circNOX4*) in A-SPZ collected from Ctrl subjects in vitro treated with vehicle or S1P ± VPC23019. RT-qPCR data were normalized using *GAPDH* expressed as fold expression (nfe) and reported as mean value ± S.E.M. Statistical significance was assessed using the two-tailed Student’s *t* test (**A**, **C**, **D**: *n* = 6 or *n* = 8 biological replicates per group) or Tukey’s post hoc-test (**E**: *n* = 6 biological replicates per group). *: *p* < 0.05, **: *p* < 0.01, a vs b *p* < 0.05.

In light of the evidence that seminal plasma EVs derived from Obese patients harbor an aberrant sphingolipid molecular architecture, we postulated that A-SPZ from the same clinical cohort might exhibit a similar profile. As delineated in Fig. 4A, a striking dysregulation of S1P metabolism and downstream signaling, almost mirroring the alterations observed in Obese EVs, was observed in sperm cells. Specifically, RT-qPCR data revealed a significant increase (*p* < 0.01) of *SPHK1, S1PR1, S1PR2, SGPP2* and *CIB1* transcripts (*p* < 0.05) in Obese compared with Ctrl A-SPZ (Fig. 4A). Conversely, a significant (*p* < 0.01) reduction of *SPHK2, SGPP1, S1PR3, S1PR4, S1PR5*, and *SPNS2* transcripts was observed in Obese A-SPZ, whereas *SGPL1* was not detected (Fig. 4A). This pattern overall highlights a compromised sphingolipid homeostasis in A-SPZ of Obese men, potentially involved in their altered functionality and increased apoptosis level.

Considering that EVs partially contribute to the sperm circRNA landscape, we hypothesized that an enhanced endogenous backsplicing activity could synergistically act with EV-mediated delivery in defining the profile of up-regulated circRNAs in Obese A-SPZ. To investigate our hypothesis, we characterized the morphological and molecular profile of FUS protein, the main RNA binding protein (RBP) reported to finely modulate endogenous backsplicing in SPZ [27, 52], by immunofluorescence and Western blot analyses, respectively. Consistent with previous data [27, 52], FUS signal was localized at the half-anterior part of sperm head and weakly in the apical area of sperm midpiece in both experimental groups, although a clear higher signal intensity was observed in Obese than Ctrl A-SPZ (Fig. 4B). In agreement with morphological data, FUS protein levels were significantly (*p* < 0.01) increased in Obese compared with Ctrl A-SPZ (Fig. 4C). In order to confirm the enhanced FUS-driven backsplicing mechanism in obesity condition, we carried out a RIP assay in Ctrl and Obese A-SPZ using FUS antibody (Fig. 4D). Relative to the use of control IgG, the results showed a significant (*p* < 0.01) fold enrichment of *cirVDAC1* (2.34 f.c.), *circTP63* (2.67 f.c.), *circXBP1* (1.87 f.c.), *circDOCK7* (2.32 f.c.) and *circNOX4* (3.12 f.c.) when the anti-FUS antibody was used in protein lysates derived Obese A-SPZ. Conversely, no FUS-bound RNA was detected for the other circRNAs investigated, demonstrating that in obesity conditions the FUS protein mediated the biogenesis of selected circRNAs likely contributing to the dysregulated molecular landscape of the sperm.

Furthermore, in view of the increase of sperm endogenous backsplicing and the deregulation of S1P signaling architecture identified in obese A-SPZ, we hypothesized that S1P axis could contribute in enhancing the endogenous FUS-dependent backsplicing observed under obesity condition. To assess this hypothesis, we designed a dedicated functional assay. Specifically, A-SPZ collected from Ctrl subjects were incubated in vitro with S1P (1 µM) ± VPC23019 (1 µM), a selective antagonist of S1P receptor type 1 (S1PR1) and 3 (S1PR3), known to be enriched in human SPZ [29]. Following the treatment, the expression levels of FUS-responsive circRNAs previously identified as upregulated in Obese A-SPZ (*circVDAC1, circTP63, circXBP1, circDOCK7, circNOX4*) were assessed by RT-qPCR. Compared to vehicle-treated control, S1P treatment induced a significant increase (*p* < 0.05) in the expression levels of all analyzed circRNAs (*circVDAC1, circTP63, circXBP1, circDOCK7, circNOX4*) (Fig. 4E). Remarkably, this effect was efficiently counteracted by co-treatment with VPC23019 antagonist (Fig. 4E), demonstrating that S1P signaling functionally modulates endogenous backsplicing, and thus supporting a mechanistic link between S1P lipidic signaling axis and FUS-dependent circRNA biogenesis.

Collectively, these data pose the critical question of whether the up-regulated circRNAs identified in Obese A-SPZ act merely as passive apoptotic markers or exert an active regulatory role in modulating the onset of sperm apoptosis.

### FUS-driven circRNA biogenesis promotes a self-reinforcing regulatory loop modulating sperm apoptosis

**Fig. 5:**
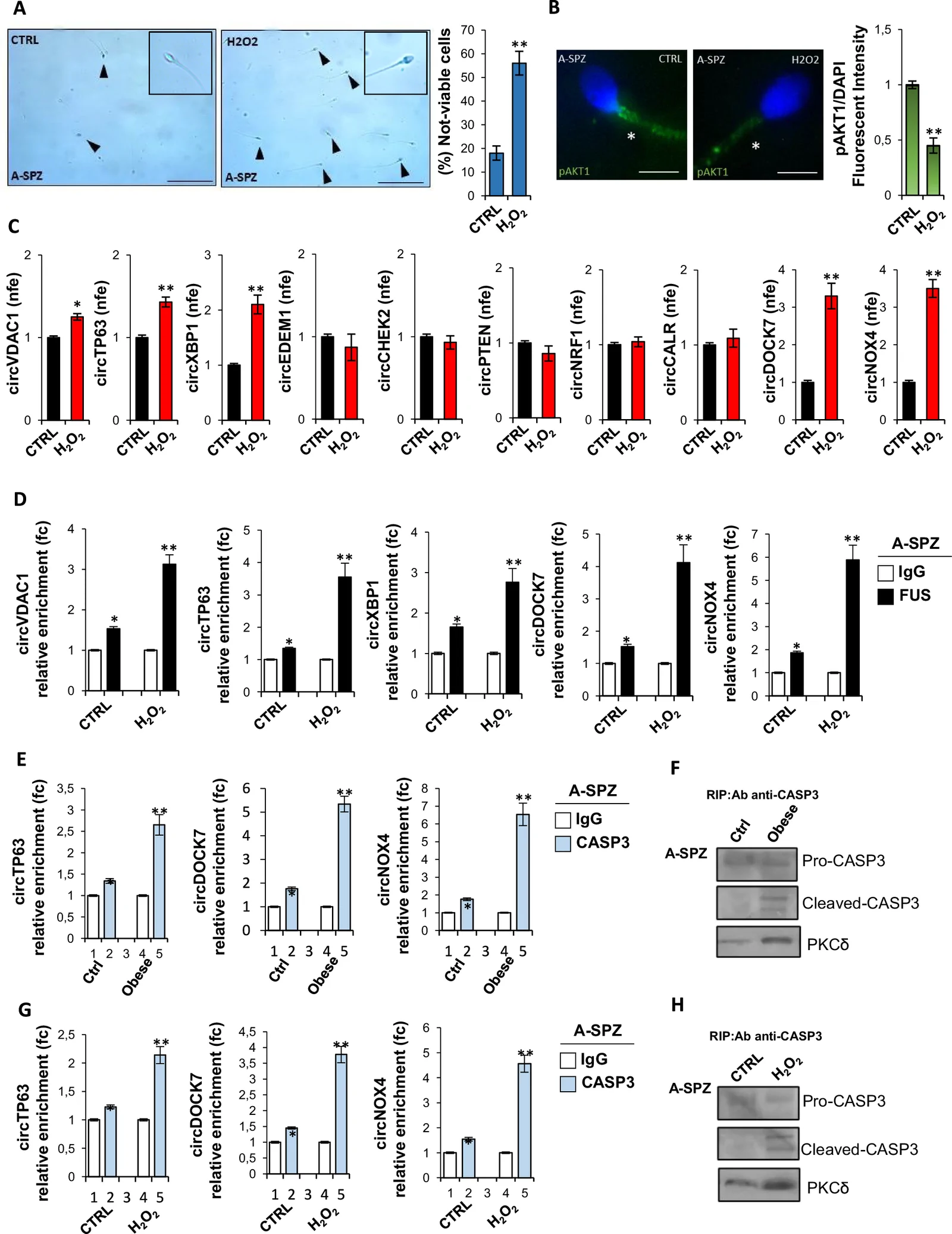
In vitro induction of sperm apoptosis promotes a circRNA-mediated pro-apoptotic regulatory loop. **A** Trypan blue dye staining of A-SPZ in vitro treated with vehicle (CTRL) or H_2_O_2_ (200 µM); black arrowheads represent Trypan blue positive sperm cells. Scale bar: 50 μm. The count of Trypan was expressed as the percentage of Trypan blue positive sperm cells/total SPZ and reported as mean ± SEM. **B** Immunofluorescence analysis and histogram showing the quantification of pAKT1 signal intensity (green) in A-SPZ in vitro treated with vehicle (CTRL) or H_2_O_2_ (200 µM). Nuclei were labeled with DAPI (blue); white asterisks represent FUS localization in sperm tail. Values were expressed as mean ± SEM; Scale bar: 4 μm. **C** Expression analysis of 10 circRNAs (*circVDAC1; circXBP1; circEDEM1; circCALR; circCHEK2; circPTEN; circTP63; circNRF1; circDOCK7; circNOX4*) in A-SPZ in vitro treated with vehicle (CTRL) or H_2_O_2_ (200 µM); RT-qPCR data were normalized using *GAPDH*, expressed as fold expression (nfe) and reported as mean value ± S.E.M. **D** The enrichment levels of 5 circRNAs (*circVDAC1; circTP63; circXBP1; circDOCK7; circNOX4*) in the products of the RIP assay (FUS-IP compared with IgG-IP) in A-SPZ in vitro treated with vehicle (CTRL) or H_2_O_2_ (200 µM) detected by RT-qPCR. Data were expressed in relative enrichment values as fold change (fc) and reported as mean ± SEM. **E**–**G** The enrichment levels of 3 circRNAs (*circTP63; circDOCK7; circNOX4*) in the products of the RIP assay (CASP3-IP compared with IgG-IP) in A-SPZ collected from Ctrl and Obese subjects (**E**) and in vitro treated (**G**) with vehicle (CTRL) or H_2_O_2_ (200 µM), detected by RT-qPCR. Data were expressed in relative enrichment values as fold change (fc) and reported as mean ± SEM. **F**–**H** Western blot analysis of CASP3 and PKCδ proteins in the immunoprecipitated products derived from the RIP assay (CASP3-IP compared with IgG-IP) performed in A-SPZ collected from Ctrl and Obese subjects (**F**) and in vitro treated (**H**) with vehicle (CTRL) or H_2_O_2_ (200 µM). Statistical differences were evaluated using the two-tailed Student’s *t* test (**A**–**D**: *n* = 6 biological replicates per group; **E**, **G**: *n* = 6 biological replicates per group); *: *p* < 0.05; **: *p* < 0.01.

To address the critical question of whether the identified up-regulated circRNAs operate merely as passive indicators of cellular stress or as active molecular drivers, we sought to characterize their clear involvement in the apoptotic cascade. Specifically, we carried out an in vitro experiment of sperm apoptosis induction and investigated their responsiveness to sperm apoptosis activation. In detail, A-SPZ were collected from Normal-weight men and in vitro treated with vehicle (CTRL) or 200 µM of H_2_O_2_, a dose previously reported to significantly promote sperm apoptosis [53, 54]. The Trypan-blue dye staining was performed to monitor the efficiency of the treatment. As reported, a significant increase (*p* < 0.01) in the percentage of Trypan-blue positive cells was observed following H_2_O_2_ treatment, demonstrating a proper apoptosis induction (Fig. 5A). To further validate the induction of the apoptotic phenotype, we carried out an immunofluorescence analysis of the anti-apoptotic pAKT1 marker (Fig. 5B). As reported, the pAKT1 signal was properly localized in sperm tail of both experimental groups, even though a weak signal intensity was detectable in H_2_O_2_ treated A-SPZ. In agreement, the fluorescence intensity analysis confirmed the significant (*p* < 0.01) down-regulation of pAKT1 signal in H_2_O_2_ when compared with CTRL A-SPZ (Fig. 5B), thus validating the experimental strategy.

In order to validate the responsiveness of spermatic circRNAs to the apoptotic pathway, the expression levels of the identified circRNAs up-regulated in Obese A-SPZ (*circVDAC1; circXBP1; circEDEM1; circCALR; circCHEK2; circPTEN; circTP63; circNRF1; circDOCK7; circNOX4*) were investigated in CTRL *vs* H_2_O_2_ treated A-SPZ by RT-qPCR analysis. The results showed a significant increase (*p* < 0.01; *p* < 0.05) of *circVDAC1, circTP63, circXBP1, circDOCK7* and *circNOX4* in H_2_O_2_ when compared with CTRL A-SPZ, whereas no effect was observed for the others investigated circRNAs (Fig. 5C), suggesting that apoptotic stimuli enhanced the endogenous backsplicing activity toward specific circRNAs.

To validate this hypothesis, and building upon the observed enhancement of endogenous FUS-dependent backsplicing observed in Obese A-SPZ, we performed a RIP assay in CTRL and H_2_O_2_ A-SPZ using FUS antibody (Fig. 5D). Relative to the use of control IgG, the results showed a significant (*p* < 0.01) fold enrichment of *cirVDAC1* (3.13 f.c.), *circTP63* (3.55 f.c.), *circXBP1* (2.76 f.c.), *circDOCK7* (4.12 f.c.) and *circNOX4* (5.89 f.c.) when the anti-FUS antibody was used in H_2_O_2_ A-SPZ. Nevertheless, for the other investigated circRNAs no FUS-bound RNA was detected, demonstrating that sperm apoptosis status promoted the FUS-mediated backsplicing only for selected circRNAs.

Finally, to verify the putative involvement of the identified FUS-mediated circRNAs (*cirVDAC1, circTP63, circXBP1, circDOCK7, circNOX4*) as molecular adapters potentially prompted to amplify the apoptotic signaling, RIP experiments were performed in A-SPZ collected from Ctrl and Obese subjects, as well as in A-SPZ in vitro treated with vehicle (CTRL) or 200 µM of H_2_O_2_, by using the anti-CASP3 antibody. Relative to the use of control IgG, the results showed a significant fold enrichment of only *circTP63* (2.65 f.c.), *circDOCK7* (5.34 f.c.) and *circNOX4* (6.54 f.c.) when the anti-CASP3 antibody was used in A-SPZ collected from Obese men (Fig. 5E), whereas no CASP3-bound RNA was detected for *circVDAC1* and *circXBP1*.

Considering that the PKCδ kinase has been reported to phosphorylate and, consequently, activate CASP3 thereby amplifying the apoptotic cascade [55, 56], we analyzed the PKCδ content in the immunoprecipitated products of RIP assay. Interestingly, an enhanced PKCδ protein signal was detected in the immunoprecipitated proteins derived from Obese A-SPZ (Fig. 5F), suggesting that circRNAs could mechanistically facilitate the physical scaffold between PKCδ and CASP3 proteins, and, in turn, the PKC-mediated CASP3 activation. In accordance with this hypothesis, an active cleaved CASP3 form was detected in the immunoprecipitated products of Obese A-SPZ (Fig. 5F), confirming an enhanced apoptotic state in this experimental group. Notably, RIP experiments carried out in in vitro H_2_O_2_ treated A-SPZ recapitulated this pattern as a significant fold enrichment of *circTP63* (2.14 f.c.), *circDOCK7* (3.78 f.c.) and *circNOX4* (4.56 f.c.) occurred in H_2_O_2_ treated A-SPZ when the anti-CASP3 antibody was used (Fig. 5G). Accordingly, Western blot analysis of relative immunoprecipitated protein counterpart showed a significant enrichment of PKCδ protein signal in H_2_O_2_ treated A-SPZ, associated with the presence of active cleaved CASP3 form (Fig. 5H), suggesting that sperm apoptotic pathway could be molecularly mediated by a putative circRNA-PKCδ-CASP3 functional axis.

## Discussion

The escalating global crisis of declining male fertility is inextricably linked to lifestyle-driven stressors that trigger sperm apoptosis [7, 27]. This alarming trend underscores a critical imperative: the discovery of novel molecular biomarkers suitable for the identification of sperm cells undergoing early apoptotic events, which severely compromise fertilization competence and the success of ART procedures [16]. CircRNAs are emerging pivotal modulators of male reproductive health [23–26, 57], thus our study sought to elucidate the contribution of these molecules in defining sperm apoptotic susceptibility. To achieve this, we exploited obesity, a hallmark lifestyle and metabolic stressor promoting sperm apoptosis [58], as a model to profile the circRNA cargo in high-quality SPZ fractions obtained from Normal-weight and Obese subjects. This experimental approach not only revealed a pro-apoptotic state in morphologically high-quality SPZ, but also served as a powerful strategy to identify selective circRNAs suitable as sensitive biomarkers of early apoptosis events. Consistent with previous reports linking obesity to (i) impaired semen quality, (ii) increased SDF index, and (iii) enhanced expression of apoptotic factors [58], our findings corroborated the presence of an enhanced pro-apoptotic phenotype in sperm cells derived from Obese subjects. The observed increase in sperm SDF index, coupled with the up-regulation of *BAX* and *CASP3* transcripts and the concomitant down-regulation of the anti-apoptotic *BCL2* one, collectively indicated that obesity shifts the equilibrium of sperm survival pathways toward programmed cell death. Alarmingly, this early apoptotic morpho-molecular signature was particularly evident within the A-SPZ fraction, as further substantiated by the decreased p-AKT1 content together with the altered SMAC and p-BAD protein profiles.

Through circRNA microarray, a total of 3920 DE-circRNAs (1914 up-regulated and 2006 down-regulated) was identified in Obese compared with Control A-SPZ. Gene ontology and KEGG pathway enrichment analyses underscored the functional relevance of these DE-circRNAs in biological processes related to metabolism, cellular response to stress, cell senescence and apoptosis. CeRNA networks suggested a functional correlation between circRNA cargo dysregulation and the activation of pro-apoptotic signaling in sperm cells, since the predicted miRNA targets (*QKI5, MAD4, SENP5, COX10, NSD1*) have been previously linked to programmed cell death [38–42].

To elucidate the regulatory circuits driving the accumulation of up-regulated circRNAs in Obese A-SPZ, the experimental validation was performed in both sperm cells and seminal plasma-derived EVs highlighting two pathways underpinning their sperm enrichment: (i) a primary testicular and/or spermatic origin, evidenced by their undetectable (*circPTEN, circNRF1*), unchanged (*circVDAC1, circTP63*) or reduced expression in EVs (*circXBP1, circEDEM1, circCHEK2*), and (ii) a supplementary vesicle-derived contribution beyond the testicular and spermatic sources (*circCALR, circDOCK7, circNOX4*). Notably, vesicle-mediated delivery only partially accounted for the overall up-regulation of circRNAs in Obese A-SPZ, thereby redirecting our focus toward the molecular characterization of the FUS protein, the principal RBP orchestrating endogenous backsplicing activity in sperm cells [52, 59]. Intriguingly, FUS was up-regulated in Obese A-SPZ and physically associated with selected circRNAs (*circVDAC1, circTP63, circXBP1, circDOCK7, circNOX4*). The central question, then, shifted: are these identified circRNAs merely molecular biomarkers or could they actively regulate and amplify the onset of sperm apoptosis? To address this, the in vitro induction of sperm apoptosis was performed by exposing A-SPZ to H₂O₂, a known inducer of this pathway [53, 54]. This exposure led to an increase in the same subset of FUS-bound circRNAs (*circVDAC1, circTP63, circXBP1, circDOCK7, circNOX4*) up-regulated in Obese sperm. Hypothesizing that these up-regulated circRNAs might represent not only passive biomarkers responsive to the apoptosis onset, but also active components of an autoregulatory circuit sustaining early sperm apoptosis onset, they might serve as molecular scaffolds able to physically bind to pro-apoptotic proteins, as previously demonstrated in other cellular models [60–63].

Interestingly, along the apoptotic cascade, beyond the classical proteolytic cleavage, numerous post-translational modifications, including the phosphorylation, can modulate the activation of caspase proteins. In this context, PKCs appear to be essential in sperm physiology [64–67]. Among them, the PKCδ isoform plays a key role in CASP3 phosphorylation [55, 56].

In response to the in vitro apoptosis induction, the RIP assay revealed that among the FUS-dependent circRNAs, only three (*circTP63, circDOCK7* and *circNOX4*) were physically associated with both the CASP3 and PKCδ proteins. This physical interaction supports the hypothesis that selected circRNAs could serve as putative molecular adapters able to promote PKCδ-dependent CASP3 activation, in turn reinforcing and amplifying the apoptotic cascade. Although this association between specific circRNAs and key components of the apoptotic machinery does not per se provide direct mechanistic evidence, it remains plausible that these circRNAs may participate in the regulation of PKCδ–CASP3 signaling, through either direct or indirect regulatory mechanisms. Thus, FUS-mediated endogenous backsplicing could simultaneously drive the biogenesis of apoptosis-responsive circRNAs and of circRNAs that, in turn, could actively modulate the apoptotic pathway. While promising, these preliminary findings require further studies to validate the inferred conclusions. Overall, the characterized molecular alterations are consistent with activation of the intrinsic mitochondrial apoptotic pathway. In particular, the imbalance between pro-apoptotic (*BAX*) and anti-apoptotic (*BCL2*) factors promotes a plausible mitochondrial outer membrane permeabilization (MOMP), a key event leading to the release of apoptogenic factors and subsequent caspase activation [68]. The concurrent modulation of SMAC and p-BAD proteins, together with the potential contribution of circRNA-mediated CASP3 activation, underscores a multilayered apoptotic network in sperm from obese subjects, integrating classical protein regulators with emerging epigenetic modulators.

In parallel, studies on bioactive sphingolipids revealed an unanticipated complexity of the lipidome and a critical role for sphingolipids in human diseases [69, 70]. S1P–to–ceramide rheostat dictates the fate of the cells [28] and altered S1P metabolism and signaling is related to metabolic syndrome [69]. Here, levels of specific long ceramide molecular species (Cer 18:0, Cer 18:1, Cer 20:0, Cer 22:0 and Cer 24:0) were found to be significantly increased in EVs from Obese patients in respect to Ctrl, according to their role in EV biogenesis [71] and release [72]. EVs are known to serve as vehicles facilitating the intercellular transport of lipids. Interestingly, exposure of astrocytes to β amyloid peptide was found to trigger the release of EVs enriched in ceramide and the pro-apoptotic protein prostate apoptosis response-4 [73] that promoted apoptosis in recipient astrocytes. The here reported data support the evidence that seminal fluid ceramide enriched EVs might induce apoptosis in recipient SPZ of Obese patients. In addition, the molecular machinery of S1P signaling was found dysregulated in both Obese EVs and SPZ. Indeed, *SPHK1*, *S1PR1* and *S1PR2* mRNA levels were significantly increased in Obese EVs while that of *SPHK2, S1PR3, S1PR5* and *SPNS2* decreased. Seminal functional studies have revealed that S1PR1 undergoes continuous activation through a constant supply of SPHK2-generated S1P within the organelles and is critical in the cargo sorting of exosomal markers such as CD63 into exosomal intraluminal vesicles [51]. Based on these findings, the dysregulation of S1P machinery in Obese EVs likely conceivably dictates altered differential cargo sorting, potentially including circRNAs. A similar dysregulated S1P signaling demonstrated in Obese A-SPZ may be attributed to the transfer of an altered machinery *via* EVs, profoundly compromising sperm capacitation and fertilization [29]. Importantly, our in vitro functional data provide direct evidence that S1P signaling can also act within SPZ to modulate the FUS-dependent endogenous backsplicing. These findings support the notion that, beyond altered EV-mediated cargo sorting, S1P signaling represents a novel intrinsic regulatory mechanism linking lipid signaling dysregulation to altered circRNA biogenesis, thereby contributing to the pro-apoptotic epigenetic signature observed in sperm from obese patients.

Collectively, sperm circRNAs and bioactive sphingolipids converge to define a molecular fault line in sperm survival. By integrating EV-mediated delivery with endogenous backsplicing activity, it is reasonable to propose that these specific circRNAs operate as both early apoptotic biomarkers and active molecular adapters that enhance apoptosis commitment.

Interestingly, perturbations in both morpho-functional and molecular parameters within the high-quality SPZ fraction have been documented under several physio-pathological conditions [59]. This observation carries profound implications for the success rate of ARTs, indicating that conventional sperm selection protocols may inadequately capture subtle early apoptotic events. Consequently, current clinical practices could significantly benefit from the implementation of complementary biomarkers to refine the assessment of sperm competence. In this scenario, *circTP63, circDOCK7* and *circNOX4* emerge as a powerful molecular blueprint, offering a novel diagnostic paradigm to predict the health, quality and hidden fertilization potential of sperm.

## Material and methods

### Ethical approval and recruitment

A total number of 120 men (age: 20–48 years) undergoing semen analysis for fertility evaluation at the Unit of Medically Assisted Reproduction, Siena University Hospital, was enrolled. Participants were classified according to body mass index (BMI) using the NHI classification into Normal-weight (18.5–24.9 kg/m²) and Obese (≥30 kg/m²) experimental groups. The study was approved by the Ethical Committee of the Siena University Hospital (approval ID CEAVSE, protocol number 25832-0-1, 02/19/2024) and written informed consent was obtained from all subjects. Individuals with known causes of male infertility (e.g., varicocele, cryptorchidism, endocrine disorders), abnormal karyotype, or a history of chemotherapy, radiotherapy, or chronic illness were excluded.

To prevent experimental bias, all samples from both experimental groups were processed and analyzed under identical, standardized laboratory conditions and data acquisition was performed in a blinded manner regarding the subjects’ clinical status.

### Sample collection and processing

Semen samples were collected by masturbation after 3–5 days of sexual abstinence and collected in sterile containers. Following complete liquefaction for 30 min at 37 °C, macroscopic and physicochemical characteristics - including pH, volume, appearance, viscosity, and liquefaction - were evaluated. Semen analysis was conducted according to WHO 2021 guidelines (ISBN:9789240030787). Briefly, 10 µL of well-mixed semen were placed in a Makler chamber to assess sperm concentration, motility, morphology, and viability. Each parameter represents the mean of two independent assessments. Sperm viability was determined using the eosin Y method. In detail, a 10 µL aliquot was mixed with 10 µL of eosin Y solution and observed under a light microscope at 400× magnification. Non-viable sperm cells appeared pink, while viable sperm cells remained unstained. All analyses were performed by experienced laboratory personnel operating under an active quality control program. After semen analysis, SPZ were collected according to standard procedures [27]. In brief, semen samples were loaded at the top of 40/80% discontinuous Puresperm gradient (Nidacon, Sweden) and centrifuged at 300 × *g* for 20 min. Following centrifugation, the seminal plasma was recovered and processed for EV isolation according to the protocol described in the corresponding section. Motile and viable SPZ were recovered from the 80% layer (A-SPZ fraction) whereas low quality SPZ were collected from the 40% layer (B-SPZ fraction). Both fractions were washed in PBS, centrifuged at 500 × *g* for 15 min, and analyzed for motility and morphology parameters. To ensure the absence of somatic cell contamination, samples were treated with Somatic Cell Lysis Buffer (0.1% SDS, 0.5% Triton X-100 in DEPC-H₂O) at 500 × *g* for 15 min and examined under light microscopy. SPZ pellets were stored at −80 °C for molecular investigations, while aliquots were dried on slides and properly stored at -20°C for morphological and immunofluorescence analysis.

### Sperm in vitro hydrogen peroxide treatment

A-SPZ samples collected from Ctrl men (*n* = 6 for each experimental group) were in vitro treated with H_2_O_2_ (Sigma, St Louis, USA) to induce sperm apoptosis, according to previous validated protocols [53, 54]. Briefly, 1 ml of sperm suspension containing 5 × 10^6^ cells was incubated with 200 μM of H_2_O_2_ and incubated at 37 °C for 2 h. Sperm suspension without the addition of H_2_O_2_ was used as CTRL experimental group. Following the treatment, the number of viable SPZ, assessed through the viable dye trypan blue reagent (trypan blue, 0.4% solution, 17-942E Lonza), was used to define the percentage of dead/total SPZ and the efficiency of the treatment. A minimum of 100 sperm cells was evaluated and counted for each analysis. All experiments were validated twice by the same operator to ensure reproducibility.

### Sperm RNA extraction

A-SPZ samples collected from Ctrl and Obese men were used for total RNA extraction by using TRIzol Reagent (Invitrogen Life Technologies, Paisley, UK) according to the manufacturer’s protocol and previously published protocol [74]. Briefly, sperm samples (5–10 × 10⁶ cells) were homogenized in 1 mL of TRIzol Reagent for 5 min at 20 °C. Subsequently, 0.2 mL of chloroform per mL of TRIzol Reagent was added to each sample, followed by centrifugation at 12,000 × *g* for 15 min at 4 °C to separate the phases. To promote RNA precipitation, the aqueous phase was mixed with isopropyl alcohol (0.5 µL per mL of TRIzol Reagent) and 1 µL of glycogen (20 mg/mL). The resulting RNA pellet was washed with 75% ethanol, centrifuged at 7500 × *g* for 10 min at 4 °C, and then dissolved in DEPC-treated water. RNA concentration (ng/µL) and purity (A₂₆₀/A₂₈₀ and A₂₆₀/A₂₃₀ ratios) were determined using a NanoDrop 2000 spectrophotometer (Thermo Fisher Scientific, Waltham, MA, USA). To eliminate residual genomic DNA, RNA aliquots (10 µg) were treated with 2 U of RNase-free DNase I (Ambion, Thermo Fisher Scientific, Waltham, MA, USA). The purified RNA samples were stored at −80 °C until further molecular analyses.

### CircRNA microarray analysis

A-SPZ samples collected from Ctrl and Obese men (*n* = 3 for each experimental group) were used for the assessment of circRNA expression profile by using a circRNA microarray analysis (Arraystar, Rockville, MD, USA). According to the Arraystar Super RNA Labeling protocol (Arraystar Inc.), enriched circRNAs were amplified and transcribed into fluorescent complementary RNA (cRNA) using a random priming method. The labeled cRNAs were then purified with the RNeasy Mini Kit (Qiagen, Germantown, MD, USA). For fragmentation, 1 μg of each labeled cRNA was mixed with 5 μL of 10× blocking agent and 1 μL of 25× fragmentation buffer, followed by incubation at 60 °C for 30 min. The fragmented and labeled cRNAs were subsequently diluted with 25 μL of 2× hybridization buffer. Hybridization was performed by dispensing 50 μL of the hybridization solution into the gasket slide and assembling the microarray slide, which was incubated for 17 h at 65 °C in an Agilent Hybridization Oven. After incubation, the slides were washed, fixed, and scanned using the Agilent Scanner G2505C (Agilent Technologies, Santa Clara, CA, USA) to obtain the fluorescent signal data.

### CircRNA profiling data and differential expression Analysis

Raw microarray images were processed using Agilent Feature Extraction Software (version 11.0.1.1) for signal acquisition and preliminary data analysis. Data normalization and further statistical processing were performed in R software (version 4.2) using the Limma package (Bioconductor). Only circRNAs detected in at least three out of six samples (flagged as “P” or “M” in “All Targets Value”) were retained for downstream analyses. Differentially expressed circRNAs (DE-circRNAs) between Ctrl A-SPZ and Obese A-SPZ were identified using *p* < 0.05 and a fold change >1.5 as significance thresholds. Throughout the text, the expression “Obese compared to Ctrl A-SPZ” refers to statistically significant DE-circRNAs identified by volcano plot filtering. Fold change filtering was applied to select DE-circRNAs, and hierarchical clustering analysis was performed to visualize distinct circRNA expression patterns between the experimental groups.

### Functional annotation of circRNA/miRNA interactions and target network construction

The predictions of circRNA–microRNA (miRNA) interactions were generated using Arraystar’s miRNA target prediction software, which integrates algorithms from both TargetScan and miRanda online analytical tools. This analysis was carried out for all DE-circRNAs to identify potential miRNA-binding sites. Validated or predicted miRNA target genes were retrieved from DIANA-TarBase v8.0 (http://www.microrna.gr/tarbase). The resulting circRNA–miRNA–target gene interaction network was constructed and visualized using the BisoGenet plug-in within the Cytoscape software platform (www.cytoscape.org). For functional annotation, the parental genes corresponding to DE-circRNAs were subjected to Gene Ontology (GO) enrichment and Kyoto Encyclopedia of Genes and Genomes (KEGG) (www.genome.jp/kegg) pathway enrichment analyses using DAVID Bioinformatics Resources 6.8 (david.ncifcrf.gov/home.jsp). Enrichment significance was assessed using the hypergeometric test, and *p*-values were corrected according to the Benjamini–Hochberg false discovery rate method. The Fold Enrichment value was considered as the enrichment score, indicating the degree of pathway association.

### CircRNA expression analysis by One-Step Evagreen RT-qPCR

The expression analysis of circRNAs in A-SPZ samples collected from Ctrl and Obese men (*n* = 8 for each experimental group), as well as in CTRL and H_2_O_2_ in vitro treated A-SPZ men (*n* = 6 for each experimental group) and EVs derived from Ctrl and Obese seminal plasma (*n* = 5 for each experimental group) was performed through One-Step Evagreen RT-qPCR reaction by using a kit containing RT-qPCR enzyme mix and an Evagreen qPCR Mastermix (Applied Biological Materials Inc., Ferndale, WA, United States), according to the manufacturer’s instructions. All reactions were performed using 50 ng of total RNA on a CFX-96 Real Time PCR System (Biorad, Milano, Italy). Assays were carried out in triplicates and included a melting curve analysis for which all samples displayed single peaks for each primer pairs. A negative control, without RNA, was also included. RNA expression was evaluated through CFX Manager software (Biorad, Milano, Italy). Reference gene stability was evaluated using RefFinder, which identified *18S* and *GAPDH* as the most stable reference genes, whereas *B2M* was the least stable. Accordingly, circRNA expression levels were normalized to *GAPDH*. Normalized fold expression (nfe) of circRNAs was calculated using the 2^−ΔΔCt^ method, and all results were expressed as mean ± SEM of nfe values.

### Linear RNA RT-qPCR analysis

The expression analysis of linear RNAs in total SPZ, as well as A-SPZ, collected from Ctrl and Obese men (*n* = 15 for each experimental group), was carried out starting from 500 ng of total RNA, which were reverse-transcribed using the High-Capacity cDNA Reverse Transcription Kit (Thermo Fisher Scientific, Waltham, MA, USA) according to the manufacturer’s instructions. Quantitative real-time PCR (RT-qPCR) was performed on a QuantStudio™ 3 Real-Time PCR System (Thermo Fisher Scientific, Waltham, MA, USA). Reactions were set up in a final volume of 10 µL containing 5 µL of PowerTrack™ SYBR™ Green Master Mix (Thermo Fisher Scientific, Waltham, MA, USA), 1 µM of each primer, and 3 µL of nuclease-free water. The amplification protocol consisted of an initial denaturation at 95 °C for 10 min, followed by 45 cycles at 95 °C for 3 s and 60 °C for annealing step. Each assay was carried out in triplicate and included a no-template negative control. Melting curve analysis was performed at the end of each run to verify primer specificity. Reference gene stability was evaluated using RefFinder, which identified *18S* and *GAPDH* as the most stable reference genes, whereas *B2M* was the least stable. Accordingly, gene expression levels were normalized to *GAPDH*. NFE was calculated using the 2^⁻ΔΔCt^ method, and all results were expressed as mean ± SEM of NFE values.

### EV isolation from seminal plasma

Seminal plasma aliquots derived from Ctrl and Obese subjects (*n* = 35 for each experimental group) were processed for EV enrichment using a combination of differential centrifugation and ultracentrifugation steps. Samples were handled on ice whenever possible and processed using sterile, RNase/DNase-free equipment; aliquots were preserved for downstream analyses. Briefly, semen samples were first centrifuged at 350 × *g* for 5 min at RT to remove cells, and the resulting supernatant was transferred to a clean tube. The supernatant then underwent sequential centrifugations at 1000 × *g* for 15 min, 2000 × *g* for 15 min, and 3000 × *g* for 15 min at 4 °C to eliminate cellular debris and larger vesicles. The clarified supernatant was subsequently passed through a 0.2 µm filter to remove residual large particles before ultracentrifugation (Ultracentrifuge Hitachi CP80NX) at 110,000 × *g* for 75 min at 4 °C, yielding a pellet enriched in small EVs. The pellet was washed with 1 ml of PBS, followed by another ultracentrifugation at 110,000 × *g* for 75 min at 4 °C. The EV-containing pellet was visually identified, and the supernatant was carefully removed by decanting, followed by gentle aspiration. The EV-containing pellet was then processed for electron microscopy or for NTA analyses or stored for RNA, protein or lipid extraction.

### EV RNA extraction

EVs collected from Ctrl and Obese seminal plasma (*n* = 10 for each experimental group) were used for total RNA extraction by using QIAzol Lysis Reagent (QIAGEN, USA) according to standard procedures. Briefly, each sample was mixed with 1 mL of QIAzol and 200 µL of chloroform and centrifuged at 12,000 × *g* for 15 min at 4 °C. The aqueous phase was transferred to a new tube, mixed with an equal volume of cold isopropanol, and incubated overnight (ON) at 4 °C, followed by centrifugation at 12,000 × g for 20 min under the same conditions. The RNA pellet was washed twice with 75% ethanol, air-dried, and resuspended in nuclease-free water. RNA concentration and purity were assessed using a NanoDrop™ One/OneC Microvolume UV–Vis Spectrophotometer (Thermo Fisher Scientific, Waltham, MA, USA).

### EV protein extraction and Western blot analysis

EV samples collected from Ctrl and Obese seminal plasma (*n* = 10 for each experimental group) were lysed using (8 M urea, 4% CHAPS, 40 mM Tris base, 65 mM dithioerythritol, and trace amounts of bromophenol blue) supplemented with Protease and Phosphatase Inhibitor (Thermo Scientific, 1861281). The samples were centrifuged at 12,000 × *g* for 20 min at 4 °C, and the soluble protein-containing supernatant was carefully collected for further analysis. Western blot analysis was carried out according to an in-house-developed protocol [75]. Briefly, the EV samples were separated by SDS-PAGE and then transferred onto nitrocellulose membrane using Power Blotter System (Invitrogen). After blocking with 5% non-fat milk in PBS, the membrane was incubated ON at 4 °C with primary antibodies (TSG-101, AB_11008389; CD81, AB_2275904; HSP70, AB_1009074). Following three washes in PBS-Tween-20 0.1%, the membranes were incubated with the appropriate with horseradish peroxidase-conjugated secondary antibody (Anti-mouse-IgG, AB_609692; Anti-Rabbit-IgG, AB_1102634) for 1 h at room temperature (RT). After three washes in PBS-Tween 20 0.1%, immunostained bands were visualized by chemiluminescence with ImageQuant LAS 4000 (GE Healthcare, Chicago, IL, USA). Alternatively, for Western blot analysis of S1P signaling components, the EV samples collected from Ctrl (*n* = 5) and Obese (*n* = 6) seminal plasma were lysed in 50 mM Tris, pH 7.5, 120 mM NaCl, 6 mM EGTA, 1 mM EDTA, 20 mM NaF, 15 mM Na_4_P_2_O_7_, 1% Nonidet, with the addition of protease inhibitor cocktail and phosphatase inhibitor cocktail. Following SDS-page separation, the proteins were transferred on PDVF membranes and incubated at 4 °C ON with different primary antibodies SPHK1 (Proteintech, Cat 10670-1-AP), SPHK2 (Proteintech, Cat 17096-1-AP), CIB1 (Proteintech, Cat 11823-1-AP), SGPL1 (Invitrogen, Cat PA1-12722), SPNS2 (Fabgennix, Cat 101-AP) S1PR1 (Proteintech, Cat 55133-1-AP), S1PR2 (Proteintech, Cat 21180-1-AP), S1PR3 (Abcam, Cat Ab126622). Subsequently, membranes were incubated with specific secondary antibodies at room temperature for 1 h. The binding of the antibodies with the specific proteins has been detected by chemiluminescence employing Amersham Imager 600 (GE Heathcare, Buckinghamshire, UK). Normalization was performed by measuring total protein directly on the membrane used for Western blot by means of stain-free technology [76] that uses a proprietary trihalo compounds in Mini Protean TGX Stain-free gels (Bio-Rad Laboratories) to enhance the fluorescence of tryptophan amino acids when exposed to UV light.

### Nanoparticle tracking analysis

Measurements of EV concentration and size were performed by NTA using a NanoSight NS 300 particle analyzer (Malvern Instruments Ltd., Malvern, UK). The EV samples collected from Ctrl and Obese seminal plasma (*n* = 6 for each experimental group) were diluted in sterile PBS, and samples were injected into the flow cell at a flow rate of 0.05 mL/s through an automated syringe pump. Five measurements (30 s/each) were obtained for each sample, and the average was plotted as a representation of size distribution and concentration (particles/mL).

### EV transmission electron microscopy

The EV samples collected from Ctrl and Obese seminal plasma (*n* = 3 for each experimental group) were prepared for transmission electron microscopy by the conventional negative staining procedure [77]. In brief, 3 μL aliquots of EV suspension were sedimented for 2 min onto a 300 mesh, copper/carbon-coated grid and then negatively stained with 1% uranyl acetate and images were taken with a TEM Fei Tecnai G2 spirit at 80 Kv.

### Liquid Chromatography-Mass Spectrometry (LC-MS/MS) Analysis

The EV samples collected from Ctrl and Obese seminal plasma (*n* = 14 for each experimental group) were resuspended in Methanol (MeOH), followed by addition of 10 µL internal standards. Samples were mixed, precipitated ON at −80 °C and then centrifugated at 21,300 × *g* for 5 min at 4 °C. The supernatant was transferred into mass spectrometry sample vials and stored at −80 °C until measurement. Analysis of sphingolipids was performed as previously described [78]. Chromatographic separation was performed on a LCMS-8050 triple quadrupole mass spectrometer (Shimadzu Duisburg, Germany) with a Dual Ion Source and a Nexera X3 Front-End-System (Shimadzu Duisburg, Germany). All MS analyses were performed using LabSolutions 5.99 SP2 and LabSolutions Insight (Shimadzu Duisburg, Germany).

### Sperm protein extraction and Western blot analysis

A-SPZ fractions collected from Ctrl and Obese men or in vitro treated with H_2_O_2_ (*n* = 6 for each experimental group) were used for total protein lysate isolation by using RIPA buffer (PBS, pH 7.4, 10 mM dithiothreitol, 0.02% sodium azide, 0.1% SDS, 1% NP-40, 0.5% sodium deoxycholate, and protease inhibitors: 10 μg/mL leupeptin, aprotinin, pepstatin A, chymostatin, and 5 μg/mL TPCK). All samples were homogenized and sonicated three times (30 s bursts at 60 mW), incubated on ice for 30 min, and centrifuged at maximum speed for 30 min at 4 °C to collect the supernatants containing total protein lysates. Protein concentrations were determined using the Lowry assay. Equal protein amounts were separated by SDS-PAGE (4–20% Mini-PROTEAN® TGX™ Precast Protein Gels; 4561094, Bio-Rad Laboratories, Milan, Italy) and transferred onto polyvinylidene difluoride (PVDF) membranes (GE Healthcare, Milan, Italy) at 280 mA for 2.5 h at 4 °C. Membranes were blocked in 5% non-fat milk with 0.25% Tween-20 in Tris-buffered saline (TBS, pH 7.6) and incubated with primary antibodies (all diluted 1:500) against AKT1 (sc-5298; Santa Cruz Biotechnology, Heidelberg, Germany), pAKT1 (sc-293125; Santa Cruz Biotechnology, Heidelberg, Germany), SMAC (sc-393118; Santa Cruz Biotechnology, Heidelberg, Germany), pBAD (sc-166932; Santa Cruz Biotechnology, Heidelberg, Germany), FUS (PA5-52610; Invitrogen, Milano, Italy), Caspase3 (PA5-77887; Invitrogen, Milano, Italy) and PKC (E-AB-14675; Elabscience). After washing with Tween-20 TBS, membranes were incubated with horseradish peroxidase-conjugated rabbit IgG (1:1000; Dako Corp., Milan, Italy) in TBS-milk buffer. Protein signals were detected using an enhanced chemiluminescence (ECL) Western Blotting Detection Reagent (Amersham RPN2106, GE Healthcare, Milan, Italy). Densitometric analysis of the immunoreactive bands was performed using Fiji (ImageJ, NIH, Bethesda, MD, USA), adjusted relative to Ponceau Red (Pon.S) and graphed in terms of optical density (OD) values as fold changes (mean ± SEM).

### RNA immunoprecipitation assay

RNA Immunoprecipitation (RIP) experiments were performed in A-SPZ collected from Ctrl and Obese men, as well as in CTRL and H₂O₂ in vitro treated A-SPZ (*n* = 6 for each experimental group) as previously described [52]. Briefly, an equal amount of total protein lysates (500 μg) was incubated with 5 μg of primary antibody (FUS, PA5-52610, Invitrogen, Milano, Italy; Caspase3, PA5-77887, Invitrogen, Milano Italy) or control IgG (12,370; Sigma-Aldrich, Milano, Italy) ON at 4 °C under rotary agitation. Subsequently, each sample was incubated for 4 h at 4 °C with 60 μL of protein A/G PLUS agarose beads (sc-2003; Santa Cruz Biotechnology, Heidelberg, Germany). After washing the beads pellets with cold TBS pH 7.6, an aliquot consisting of 10% of total beads was removed before RNA isolation from each sample for the following immunoprecipitated protein analysis by Western blot. Total RNAs were extracted using TRIzol Reagent (Invitrogen Life Technologies, Paisley, UK). Finally, a NanoDrop 2000 spectrophotometer (Thermo Fisher Scientific, Waltham, MA, USA) was used to quantify the immunoprecipitated RNAs with FUS and control IgG. The immunoprecipitated RNAs were stored at −80 °C for molecular investigations.

### Vesicle shuttle in vitro experiment

A-SPZ collected from Ctrl men (*n* = 6) were separately incubated for 30 min at 37 °C in 1 mL of: (i) PBS (pH 7.6), (ii) Obese-derived seminal plasma (Obese-SP) or (iii) Obese-SP pretreated for 2 h at 37 °C with anti-CD9 antibody (sc-13118; Santa Cruz Biotechnologies, Heidelberg, Germany), accordingly to previous validated protocols [52]. After treatment, SPZ were centrifuged at 1500 × *g* for 20 min at 4 °C, washed in PBS, and stored at −80 °C for molecular investigations.

### Sperm in vitro sphingosine-1-phosphate treatment

The Sphingosine 1-Phosphate (S1P; Merck; #567727) and the selective antagonist of S1PR1 and S1PR3 receptors (VPC23019; Cayman Chemicals; #13240) were used for sperm in vitro treatments. The compounds were dissolved in dimethylsulfoxide (DMSO) and BSA, respectively, according to the manufacturer’s instructions. A-SPZ collected from Ctrl men (*n* = 6) were separately incubated with 1 mL of PBS with the vehicle (according to relative compound concentrations; control vehicle group) or in S1P (1 μM) ± VPC23019 (1 μM), for 30 min at RT. The VPC23019 was added 5 min before S1P at a concentration useful to affect S1P activity, as previously reported in cell culture systems [79]. After treatment, SPZ were centrifuged at 1500 × *g* for 20 min at 4 °C, washed in PBS, and stored at −80 °C for molecular investigations.

### Tunel assay

SDF in total SPZ, as well as in A-SPZ collected from Ctrl and Obese men (*n* = 15 for each experimental group), was assessed using the TUNEL assay (DeadEnd™ Fluorometric TUNEL System, Promega, USA) following the manufacturer’s instructions and previously described [74]. Briefly, semen samples were washed with DPBS and centrifuged at 600 × *g* for 10 min. The pellet was resuspended in DPBS at a concentration of 10 × 10⁶ sperm/mL, smeared onto slides, and fixed in 4% paraformaldehyde for 25 min. After washing with 1% BSA/DPBS, samples were permeabilized with 0.2% Triton® X-100 for 5 min and washed twice. Slides were then incubated with equilibration buffer for 10 min at RT, followed by the reaction mixture (rTdT enzyme, nucleotide mix, and equilibration buffer) at 37 °C for 1 h. After incubation, slides were washed in 2× SSC for 15 min and three times (5 min each) in 1% BSA/DPBS to remove unincorporated fluorescein-12-UTP. Nuclei were counterstained with DAPI and mounted with DABCO. Images were acquired using Leica AF6500 Integrated System for Imaging and Analysis (Leica Microsystems, Wetzlar, Germany) equipped with the LAS software. SPZ were classified as TUNEL⁻ (blue, non-fragmented) or TUNEL⁺ (bright green, fragmented). For each sample, at least 200 SPZ were evaluated in randomly selected microscopic fields, and the percentage of TUNEL⁺ cells was calculated as an index of SDF.

### Annexin-V immunofluorescence assay

Early apoptosis was evaluated in Total- and A-SPZ fractions from normozoospermic men (*n* = 6) using the FITC Annexin V/Dead cell Apoptosis Assay Kit (Invitrogen Ltd., Renfrew, UK), following the manufacturer’s instructions. The SPZ fractions were washed with PBS, centrifuged at 400 × *g* for 5 min and resuspended in Annexin Binding Buffer (ABB) to a final concentration of 1 × 10⁶ cells/mL. For each sample, 5 µL of Annexin V conjugated with fluorescein isothiocyanate (Annexin V-FITC) were added, followed by incubation for 15 min at RT. Sperm cells were then gently washed with ABB, and a drop of the suspension was smeared onto glass slides. Slides were mounted using DABCO (Sigma-Aldrich, Milan, Italy). Fluorescence observations were performed using a Leica DMi8 fluorescence microscope (Leica Microsystems, Wetzlar, Germany). For each sample, 200 sperm cells were analyzed. Green fluorescence indicates early apoptotic cells. All experiments were conducted in triplicate and results are expressed as the percentage of early apoptotic SPZ.

### Immunofluorescence analysis

Immunofluorescence analysis was performed in A-SPZ collected from Ctrl and Obese men, as well as in CTRL and H₂O₂ in vitro treated A-SPZ (*n* = 6 for each experimental group). SPZ were air-dried on slides, fixed in 4% paraformaldehyde (sc-281692; Santa Cruz Biotechnology, Heidelberg, Germany) for 20 min at RT, and permeabilized with 0.1% Triton X-100 (X100; Sigma-Aldrich, Milan, Italy) in PBS for 10 min at RT. Blocking was carried out using 10% bovine serum albumin (05470; Sigma-Aldrich, Milano, Italy) for 30 min at RT. Then SPZ were incubated ON at 4 °C with primary antibodies (all diluted 1:100) against pAKT1 (sc-293125; Santa Cruz Biotechnology), SMAC/DIABLO (sc-393118; Santa Cruz Biotechnology), and FUS (PA5-52610; Invitrogen, Milan, Italy). Following three washes in Dulbecco’s PBS (DPBS, 1×), slides were incubated with secondary antibodies conjugated to FITC (711-095-152 or 715-095-150; Jackson ImmunoResearch, Cambridge, UK) diluted 1:200 for 1 h at 37 °C. Nuclei were counterstained with DAPI (D9542; Sigma-Aldrich, Milan, Italy). Samples were examined using a Leica DM 5000 B + CTR 5000 optical microscope (Leica Microsystems, Wetzlar, Germany) equipped with a UV lamp. Images were captured with a Leica DFC320 R2 digital camera and analyzed using IM1000 software (version 4.7.0; Leica Microsystems, Wetzlar, Germany). All experiments were validated twice by the same operator to ensure reproducibility.

### Primer design

Primers used to validate and amplify selected mRNAs and circRNAs in human SPZ were designed using the Primer-BLAST online tool (http://www.ncbi.nlm.nih.gov/tools/primer-blast/). To ensure specificity for circRNA isoforms, primers were designed to span the back-splicing junction of each target circRNA. In addition, specific primers were designed for the housekeeping genes used as an internal reference for normalization. All primer sequences are listed in Table 1.

**Table 1:** Primer sequences used in RT-qPCR.

Gene primers	Sequences 5′-3′
*GAPDH S*	ACATCGCTCAGACACCATG
*GAPDH AS*	TGTAGTTGAGGTCAATGAAGGG
*B2M S*	GGACTGGTCTTTCTATCTCTTGTAC
*B2M AS*	ACCTCCATGATGCTGCTTAC
*18S S*	GGGAGGTAGTGACGAAAAATAAC
*18S AS*	TACTGGCACCACTGGAAACC
*BCL2 S*	GGCTGGGATGCCTTTGTG
*BCL2 AS*	GCCAAACTGAGCAGAGTCTTCA
*CASP3 S*	Hs.PT.56a.25882379.g
*CASP3 AS*	
*SPHK1 S*	ATAAGGAGCTGAAGGCAGGA
*SPHK1 AS*	CATCAGCGTGAAGGAGATTT
*SPHK2 S*	GAAGCTGTGAAGATGCCTGT
*SPHK2 AS*	CCACAGACAGGAAGGAGAAA
*SGPL1 S*	ACAGTGTACAGTGGGGAGGA
*SGPL1 AS*	GAAGTCACACATCCACACGA
*SGPP1 S*	CCCATTTCTATGGTCCTCCT
*SGPP1 AS*	TTGTCAATCAGGTCCACAAAT
*SGPP2 S*	CCAAGGATGTCTTGAAGTGG
*SGPP2 AS*	AGACACACCAAGGTGGAAAA
*S1PR1 S*	AACCAGTGGTTGAAGACTAGCAATGGTC
*S1PR1 AS*	TTCCAGATGCGTTCACCAGATAGC
*S1PR2 S*	TCGTAGCCAATACCTTGCTC
*S1PR2 AS*	CTGCCATACAGCTTGACCTT
*S1PR3 S*	GACCATCGTGATCCTCTACG
*S1PR3 AS*	GAACCACTGAGCCTTGAAGA
*S1PR4 S*	TTCTGCACTACAACCACTCG
*S1PR4 AS*	AGGTCACTCAGCGTGATGTT
*S1PR5 S*	GGGAAGACCAAAAGAAAAGC
*S1PR5 AS*	CTCAGAGCAAAAGCGAAGTC
*SPNS2 S*	ACGTGCTCAACTACCTGGAC
*SPNS2 AS*	ATGAAGGAGCTGGAGAAGGT
*circVDAC1 S*	TAGGCACCGAGATTACTGTGG3
*circVDAC1 AS*	CCATCTTCTGCCAGTGTTAGGT
*circXBP1*	AAGGTACAATGCAGGCGCT
*circXBP1 AS*	CCACCACCATAGCTCCAGAC
*circEDEM1 S*	ATCTCCTCTACCAGGTGCTGAT
*circEDEM1 AS*	CTCTCAGGGAGGGCACCATA
*circCALR S*	GGGTCCCAGATTGGCTCACA
*circCALR AS*	CGCCGCGGAGACACAA
*circPTEN S*	TCGGGGCAAATTTTTAAAGGCA
*circPTEN AS*	
*circCHEK2 S*	TTGGTTCAGCAAGAGAGGCA
*circCHEK2 AS*	TGAGCCTCAACATCCGACT
*circTP63 S*	TGACCCCATGTGGTTTCGTAG
*circTP63 AS*	TTGTCTGTGTGCTCTGGGAC
*circNRF1 S*	GGCCACAGCCACACATAGTA
*circNRF1 AS*	TGGGTCCATGAAACCTGGATAA
*circDOCK7 S*	TGCTTCAGAATGGACGGTTG
*circDOCK7 AS*	AGAGAAGATTTCTCAGGAGACAGTA
*circNOX4 S*	TCACAATGAGGGCTGCTGAA
*circNOX4 AS*	TCTGAGAGCTGGTTCGGTTAAG
*circSMG1 S*	GCAACAGTTCAGGCGTAACC
*circSMG1 AS*	CGCTCATACTGCAGAGTTCC
*circBAX S*	ACCCTCTGACCCTAGCTCTT
*circBAX AS*	TCAGCTGCCACTCGGAAAAA
*circATF6 S*	TCAGCATGTTCCTATTCTGCTCT
*circATF6 AS*	GCTGCTTCCAATTGCAGCTC
*circDIABLO S*	TGCATATCAAACTGGTTCCTGC
*circDIABLO AS*	ATAAGGCACGACCGCCAATC
*circHDAC6 S*	CCCAGGCCTCCTGGACAT
*circHDAC6 AS*	CGGATGCGGTGTTTCTGTTG
*circPUM1 S*	ATCCTTATGGCTGCCGAGTG
*circPUM1 AS*	TGCCAAACTGTACAAGCTGC
*circATG7 S*	CATTTGCAGAGTGCTCCCAC
*circATG7 AS*	AGACTCGAGTGTGTTGGTGT
*circFOXO1 S*	GAGGGTTAGTGAGCAGAATTCAA
*circFOXO1 AS*	TGGATTGAGCATCCACCAAGA
*circRBM5 S*	CGGCTGTAGTGTCCCAGAGT
*circRBM5 AS*	GCTGAGAAGCATCCGGAGAG
*circSIRT7 S*	CCCTCTGAACCATCCCATCC
*circSIRT7 AS*	GCAAGGGAATCACAAAGGGGA

### Statistical analysis

Sample size formal justification and its multi-phase allocation workflow are detailed below. Briefly, for the high-throughput Discovery Phase, a sample size of *n* = 3 biological replicates per group was selected based on established international standards for circRNA microarray profiling. For downstream validation and expanded characterizations, sample sizes were progressively scaled up to meet specific technical requirements and biological validation tiers (*n* = 8 for RT-qPCR, *n* = 15 for linear RNA and TUNEL assays), and up to *n* = 35 per group for clinical and phenotypic characterizations (e.g., seminal plasma EV profiling, lipid signaling quantification).

To guarantee an unbiased outcome assessment, a blinded approach was implemented during the experimental and analytical workflows; all biological samples were assigned unique alphanumeric codes upon collection, masking the donors’ clinical status from the investigators during sample processing, data acquisition, and quantification. Based on a post-hoc sensitivity power analysis (*β* = 0.80, *α* = 0.05, two-tailed), these sample sizes provide adequate statistical power to detect minimum effect sizes ranging from Cohen’s *d* = 1.50 (for *n* = 8) down to *d* = 0.68 (for *n* = 35). The normality of data distribution and the homogeneity of variance were assessed using the Shapiro–Wilk and Levene’s tests, respectively. Intra-group data variation and dispersion were systematically calculated for each experimental group, and differences between two independent groups were evaluated using the Student’s *t* test or the Mann–Whitney *U* test, depending on normality distribution, whereas multiple comparisons were analyzed *via* one-way ANOVA followed by Tukey’s post-hoc test. Differences with *p* < 0.05 were considered statistically significant. All data were presented as mean ± SEM.

## Supplementary information

Supplemental Figure legends

Supplemental Figure 1

Supplemental Figure 2

Supplemental Figure 3

Supplemental Figure 4

Supplemental Figure 5

Uncropped blots

## Data Availability

The datasets used and/or analyzed during the current study are available from the corresponding author on reasonable request.
