# Supplementary material for: The sperm survival switch: circRNA-mediated regulatory loops control apoptotic signalling in human spermatozoa
Source: Cell Death Discov. 2026 Jul 25;12:362. doi: 10.1038/s41420-026-03262-0 (PMC13562583; doi:10.1038/s41420-026-03262-0)

A

B

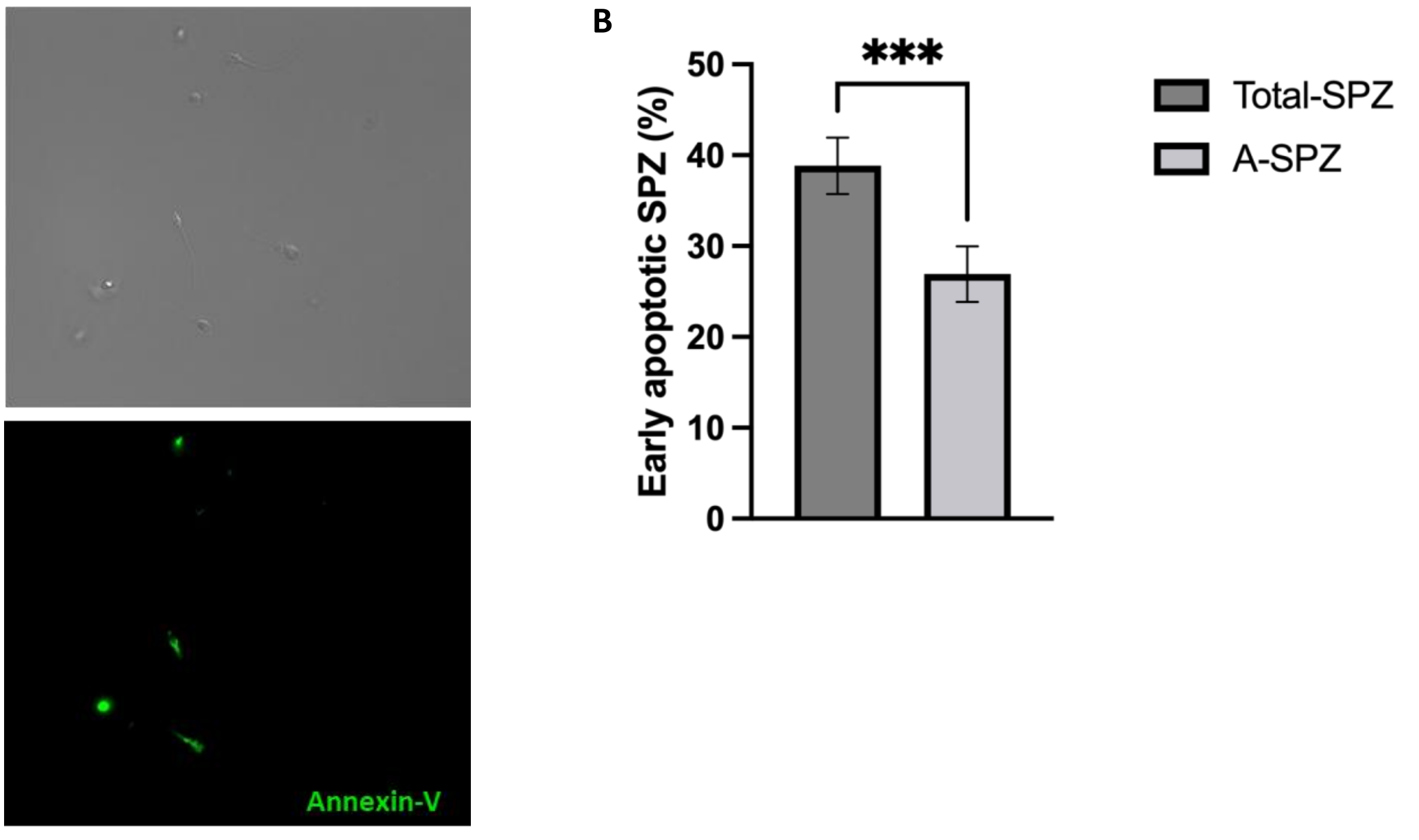

Supplement: Supplementary file 1 [file 41420_2026_3262_MOESM2_ESM.pdf]
