# Supplementary material for: The sperm survival switch: circRNA-mediated regulatory loops control apoptotic signalling in human spermatozoa
Source: Cell Death Discov. 2026 Jul 25;12:362. doi: 10.1038/s41420-026-03262-0 (PMC13562583; doi:10.1038/s41420-026-03262-0)
Supplement: Supplementary file 2 [file 41420_2026_3262_MOESM3_ESM.pdf]

A

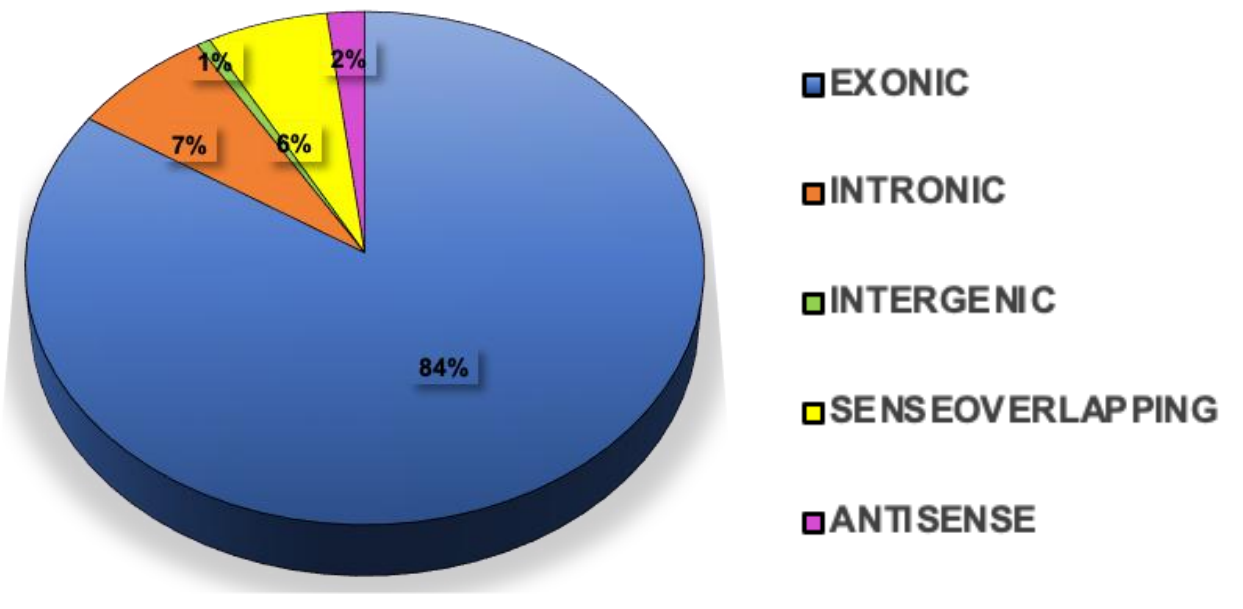
1%
2%
7%
6%
84%
EXONIC
INTRONIC
INTERGENIC
SENSEOVERLAPPING
ANTISENSE

B

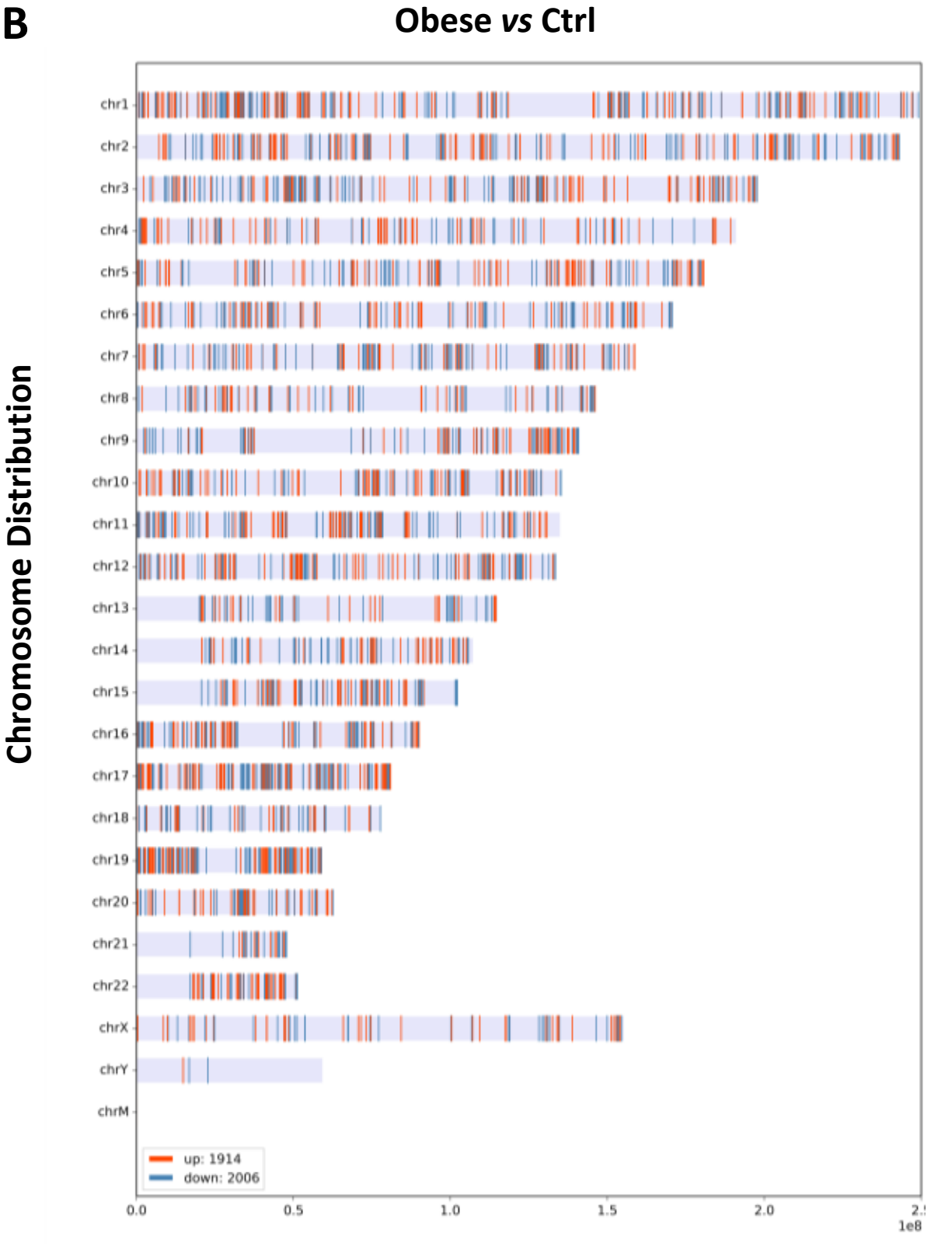
Obese vs Ctrl
Chromosome Distribution
chr1
chr2
chr3
chr4
chr5
chr6
chr7
chr8
chr9
chr10
chr11
chr12
chr13
chr14
chr15
chr16
chr17
chr18
chr19
chr20
chr21
chr22
chrX
chrY
chrM
up: 1914
down: 2006
0.0
0.5
1.0
1.5
2.0
1e8
