# Supplementary material for: The sperm survival switch: circRNA-mediated regulatory loops control apoptotic signalling in human spermatozoa
Source: Cell Death Discov. 2026 Jul 25;12:362. doi: 10.1038/s41420-026-03262-0 (PMC13562583; doi:10.1038/s41420-026-03262-0)
Supplement: Supplementary file 3 [file 41420_2026_3262_MOESM4_ESM.pdf]

A

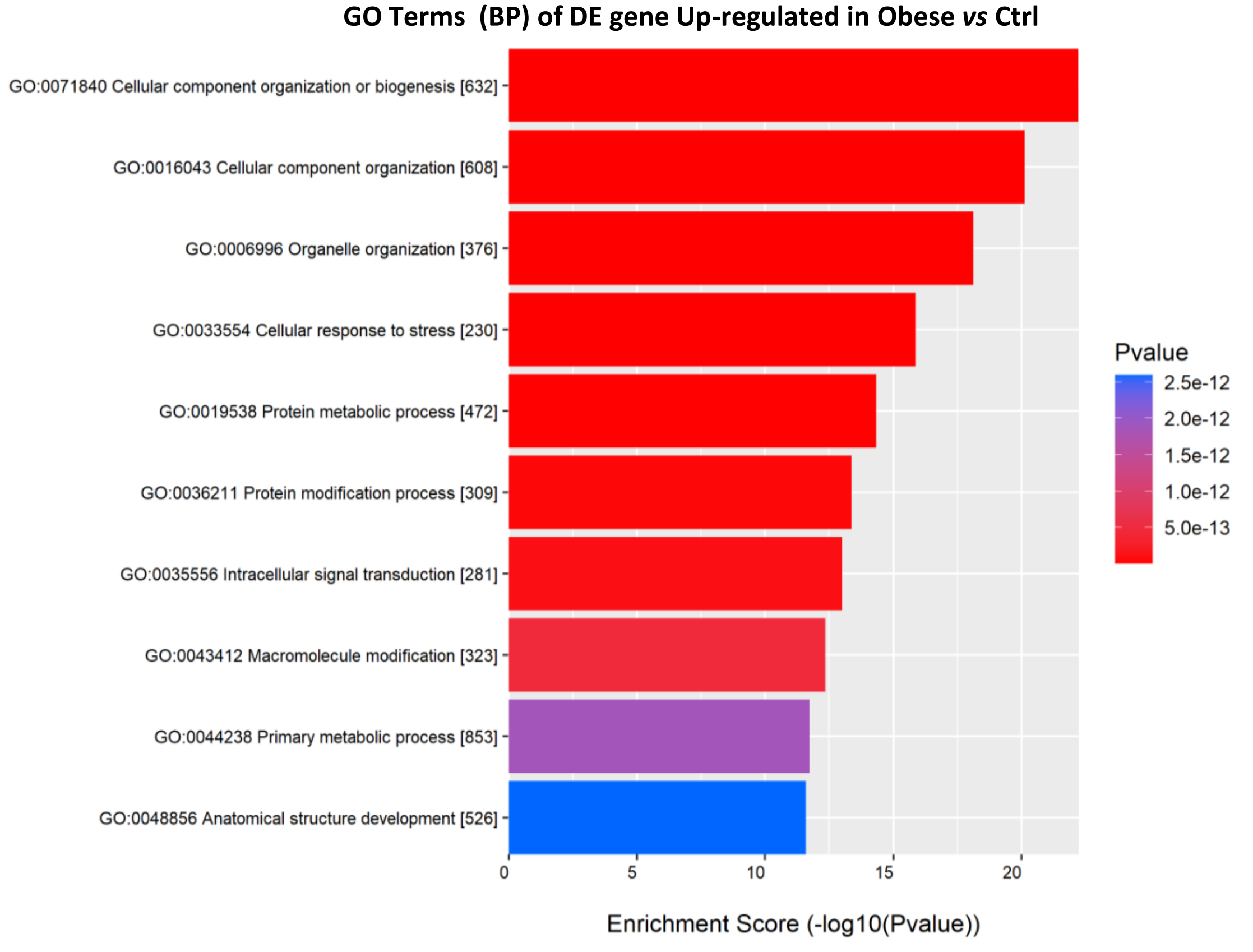
GO Terms (BP) of DE gene Up-regulated in Obese vs Ctrl
GO:0071840 Cellular component organization or biogenesis [632]
GO:0016043 Cellular component organization [608]
GO:0006996 Organelle organization [376]
GO:0033554 Cellular response to stress [230]
GO:0019538 Protein metabolic process [472]
GO:0036211 Protein modification process [309]
GO:0035556 Intracellular signal transduction [281]
GO:0043412 Macromolecule modification [323]
GO:0044238 Primary metabolic process [853]
GO:0048856 Anatomical structure development [526]
0
5
10
15
20
Enrichment Score (-log10(Pvalue))
Pvalue
2.5e-12
2.0e-12
1.5e-12
1.0e-12
5.0e-13

B

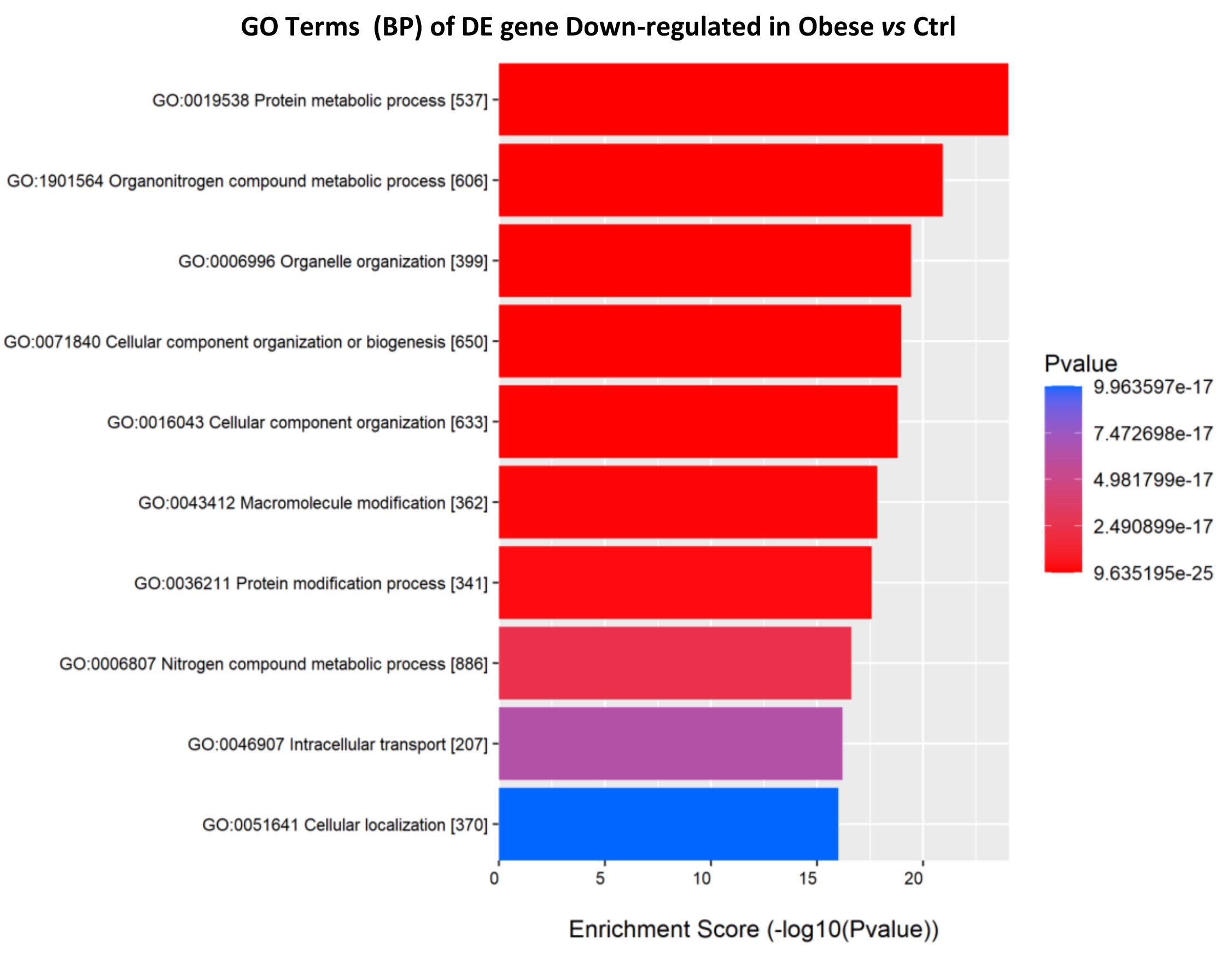
GO Terms (BP) of DE gene Down-regulated in Obese vs Ctrl
GO:0019538 Protein metabolic process [537]
GO:1901564 Organonitrogen compound metabolic process [606]
GO:0006996 Organelle organization [399]
GO:0071840 Cellular component organization or biogenesis [650]
GO:0016043 Cellular component organization [633]
GO:0043412 Macromolecule modification [362]
GO:0036211 Protein modification process [341]
GO:0006807 Nitrogen compound metabolic process [886]
GO:0046907 Intracellular transport [207]
GO:0051641 Cellular localization [370]
0
5
10
15
20
Enrichment Score (-log10(Pvalue))
Pvalue
9.963597e-17
7.472698e-17
4.981799e-17
2.490899e-17
9.635195e-25
