# Supplementary material for: The sperm survival switch: circRNA-mediated regulatory loops control apoptotic signalling in human spermatozoa
Source: Cell Death Discov. 2026 Jul 25;12:362. doi: 10.1038/s41420-026-03262-0 (PMC13562583; doi:10.1038/s41420-026-03262-0)
Supplement: Supplementary file 4 [file 41420_2026_3262_MOESM5_ESM.pdf]

A

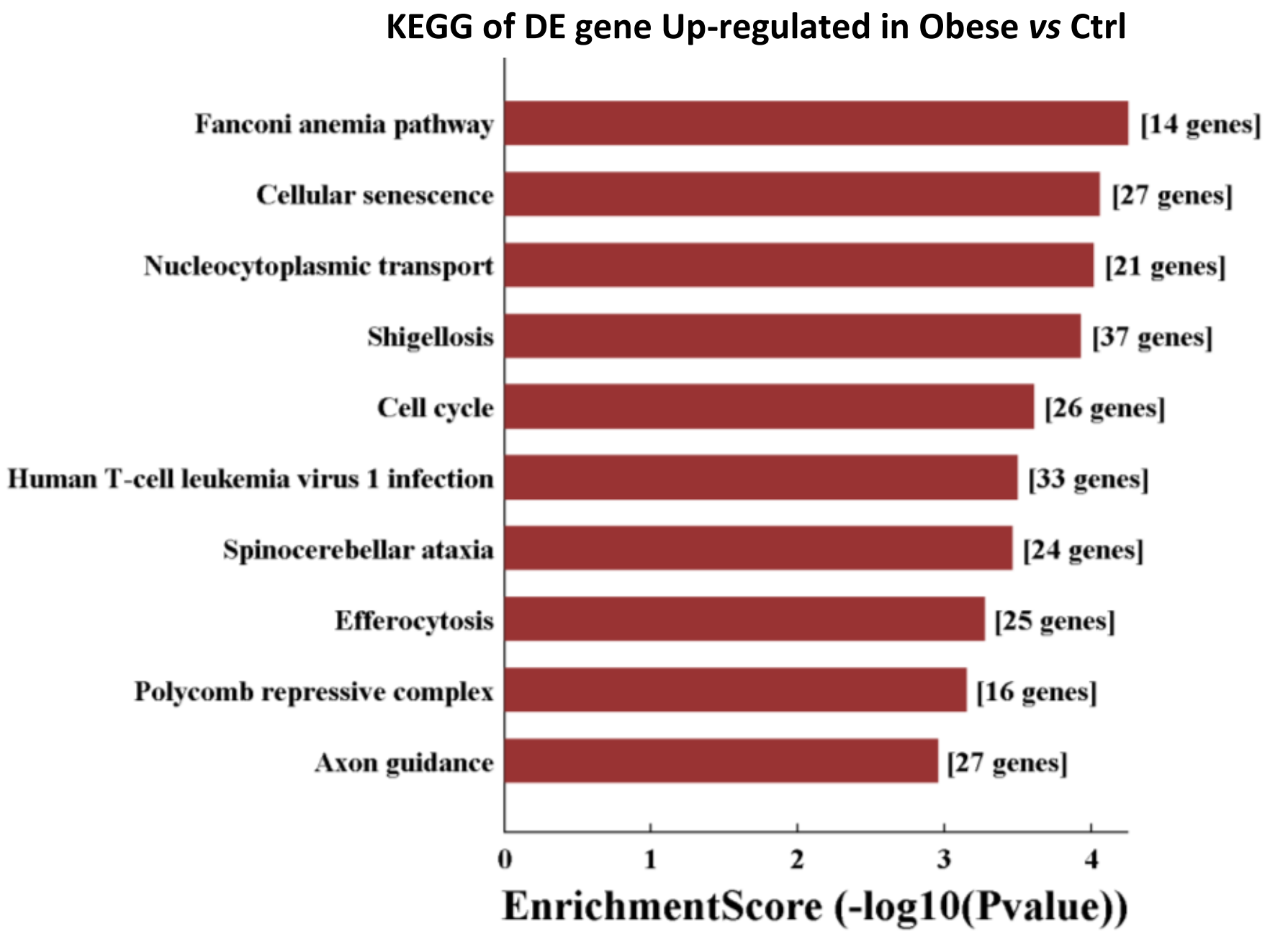
KEGG of DE gene Up-regulated in Obese vs Ctrl
Fanconi anemia pathway
Cellular senescence
Nucleocytoplasmic transport
Shigellosis
Cell cycle
Human T-cell leukemia virus 1 infection
Spinocerebellar ataxia
Efferocytosis
Polycomb repressive complex
Axon guidance
[14 genes]
[27 genes]
[21 genes]
[37 genes]
[26 genes]
[33 genes]
[24 genes]
[25 genes]
[16 genes]
[27 genes]
0
1
2
3
4
EnrichmentScore (-log10(Pvalue))

B

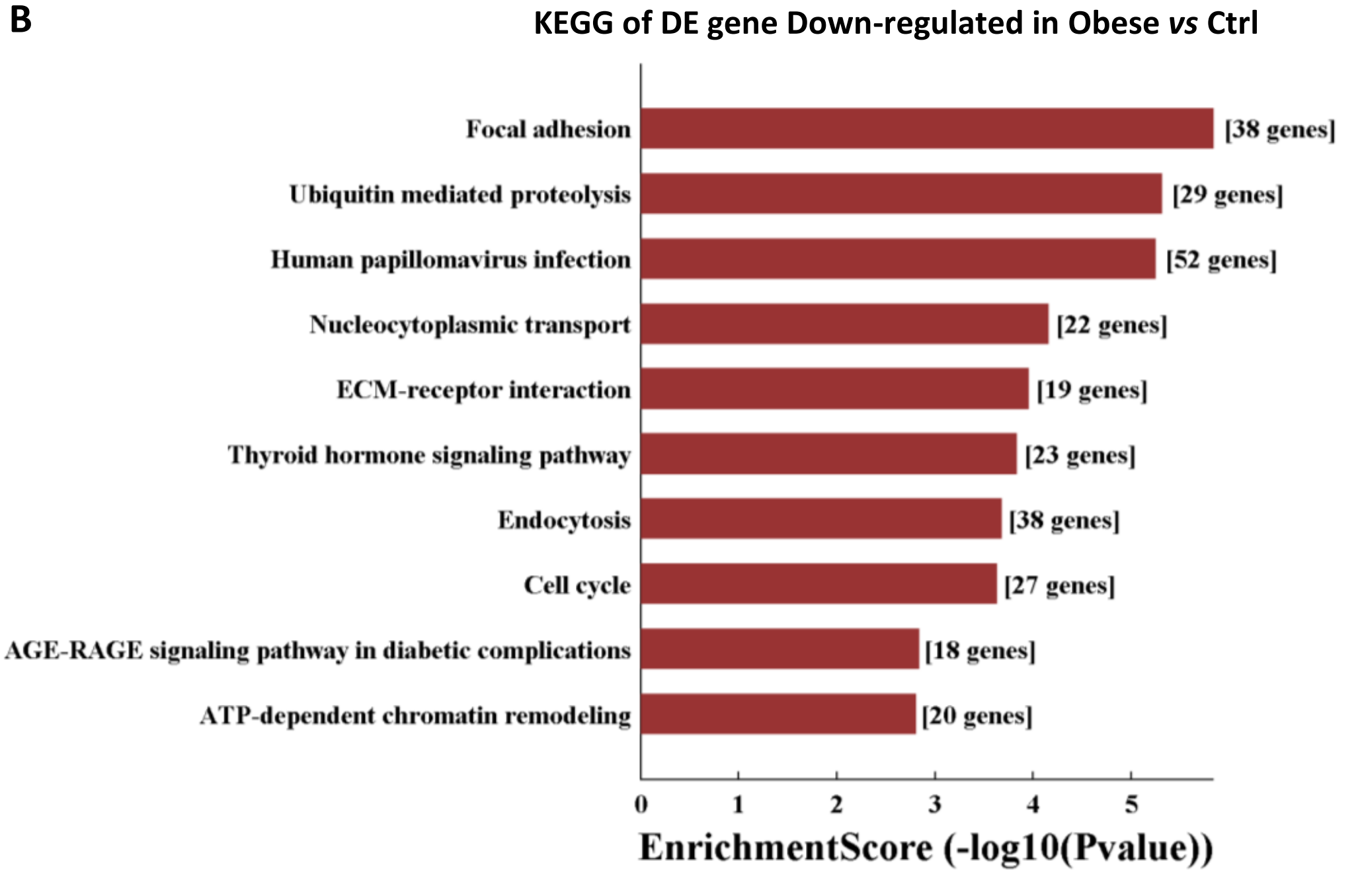
KEGG of DE gene Down-regulated in Obese vs Ctrl
Focal adhesion
Ubiquitin mediated proteolysis
Human papillomavirus infection
Nucleocytoplasmic transport
ECM-receptor interaction
Thyroid hormone signaling pathway
Endocytosis
Cell cycle
AGE-RAGE signaling pathway in diabetic complications
ATP-dependent chromatin remodeling
[38 genes]
[29 genes]
[52 genes]
[22 genes]
[19 genes]
[23 genes]
[38 genes]
[27 genes]
[18 genes]
[20 genes]
0
1
2
3
4
5
EnrichmentScore (-log10(Pvalue))
