# Supplementary material for: The sperm survival switch: circRNA-mediated regulatory loops control apoptotic signalling in human spermatozoa
Source: Cell Death Discov. 2026 Jul 25;12:362. doi: 10.1038/s41420-026-03262-0 (PMC13562583; doi:10.1038/s41420-026-03262-0)
Supplement: Supplementary file 5 [file 41420_2026_3262_MOESM6_ESM.pdf]

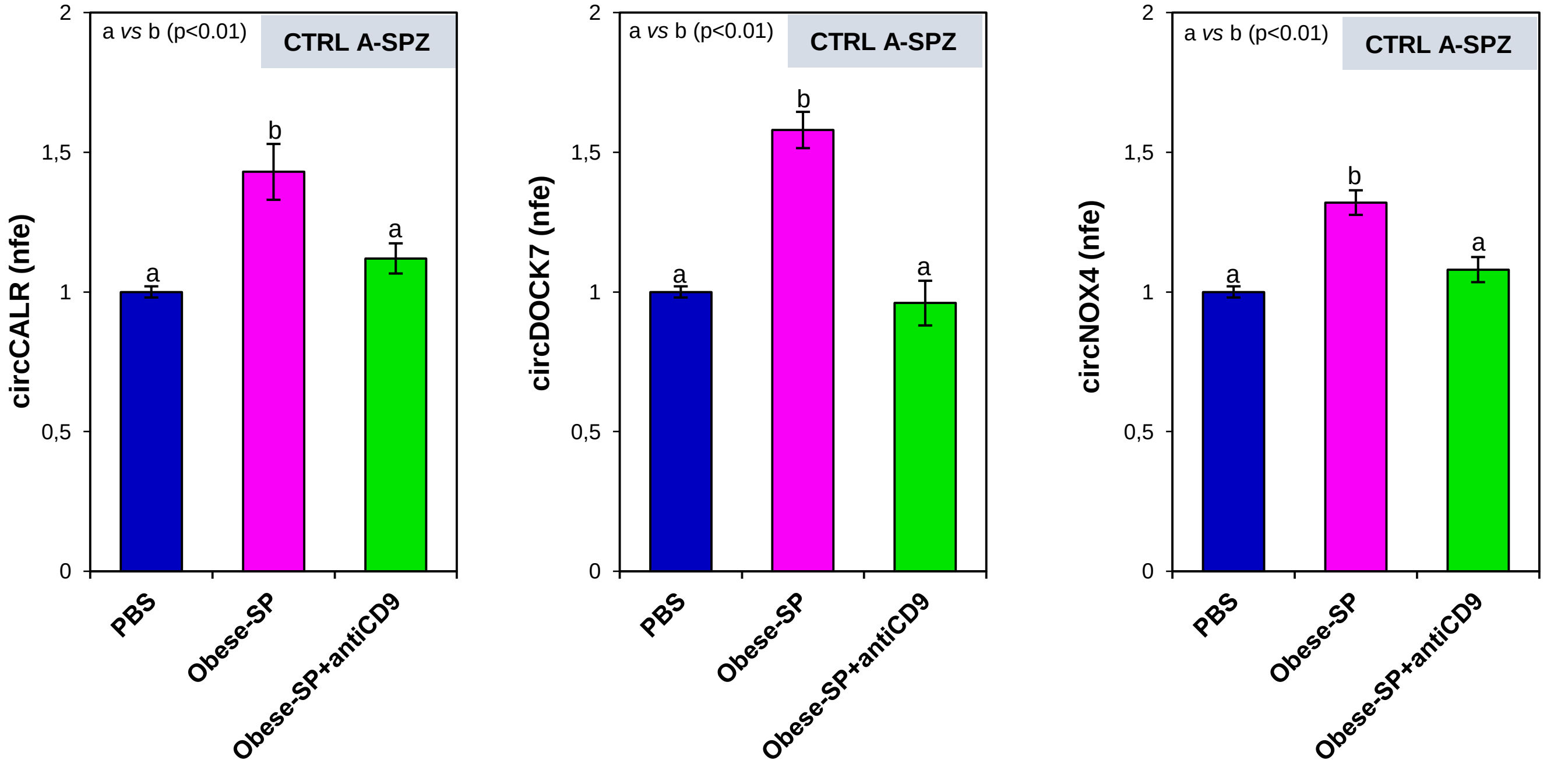

a vs b (p<0.01)
CTRL A-SPZ
circCALR (nfe)
circDOCK7 (nfe)
circNOX4 (nfe)
0
0,5
1
1,5
2
a
b
a
PBS
Obese-SP
Obese-SP+antiCD9
