# Supplementary material for: The sperm survival switch: circRNA-mediated regulatory loops control apoptotic signalling in human spermatozoa
Source: Cell Death Discov. 2026 Jul 25;12:362. doi: 10.1038/s41420-026-03262-0 (PMC13562583; doi:10.1038/s41420-026-03262-0)
Supplement: Supplementary file 6 [file 41420_2026_3262_MOESM7_ESM.pdf]

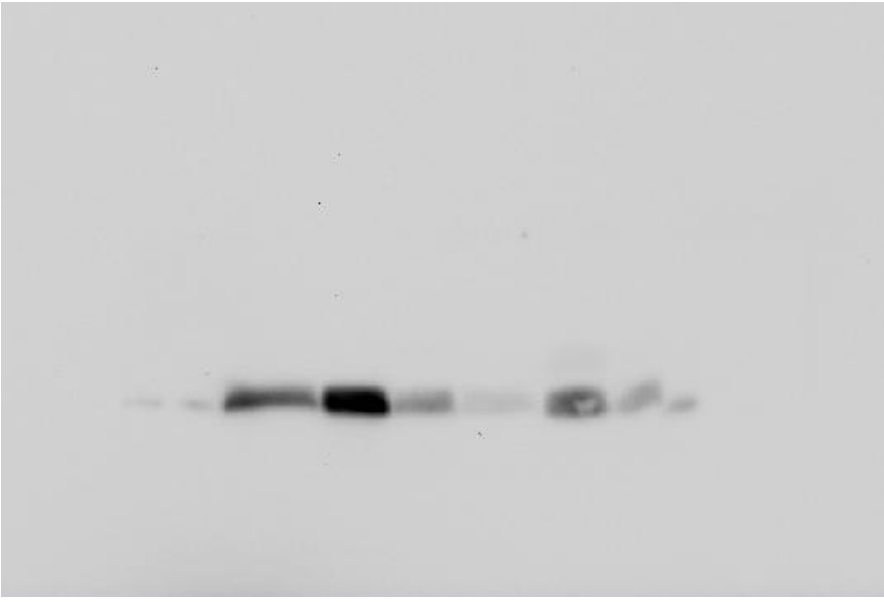
CD81

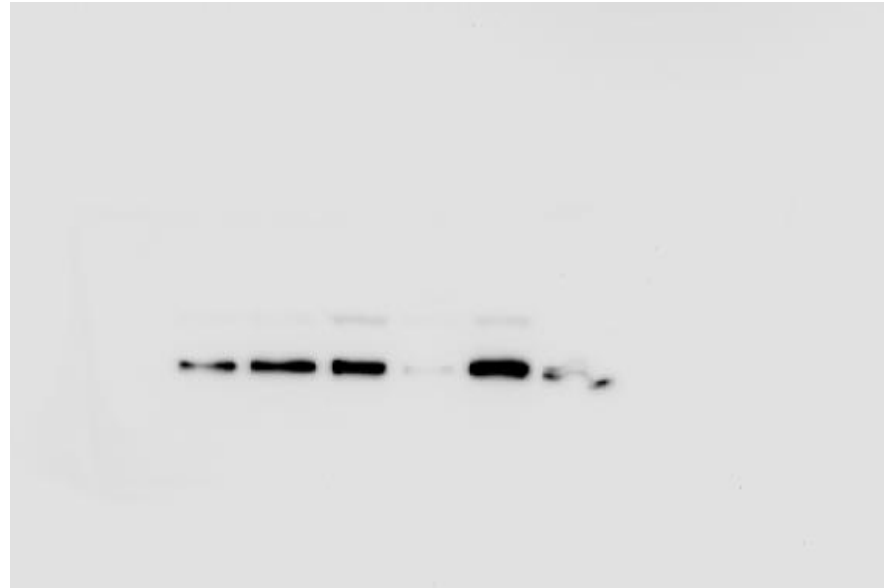
HSP70

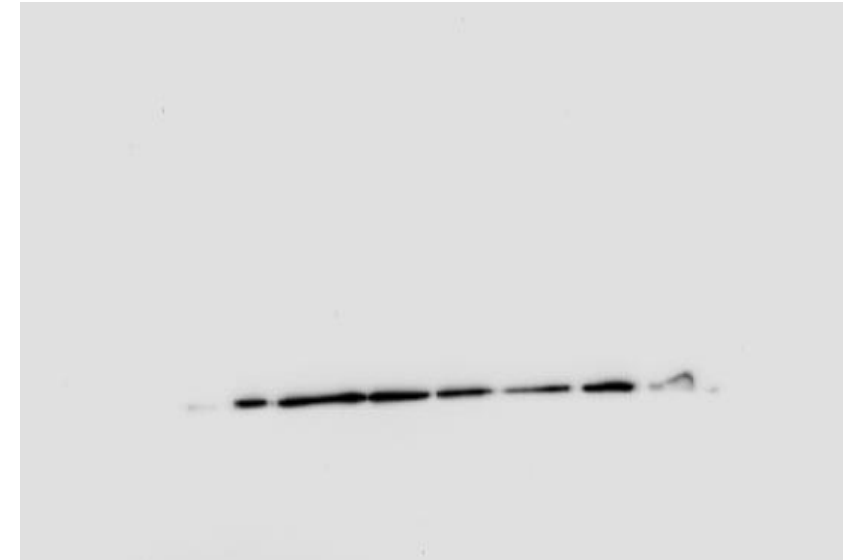
TSG101

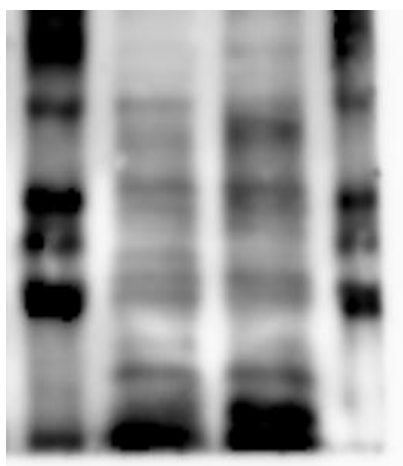
SPHK1

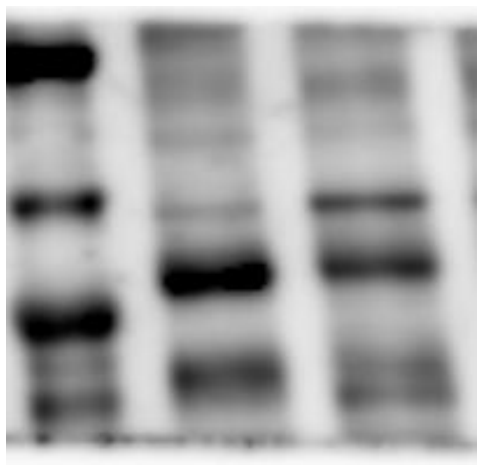
SPHK2

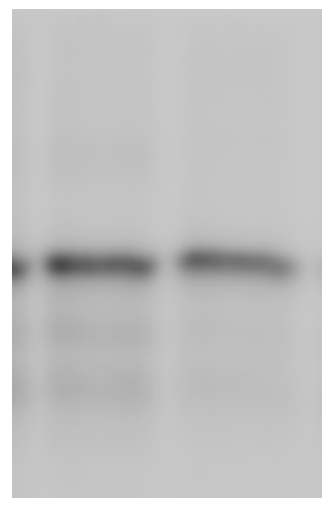
CIB1

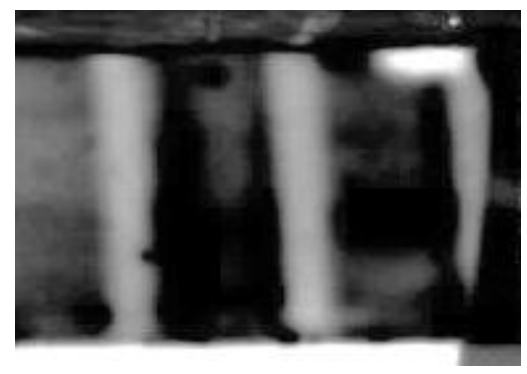
SGPL1

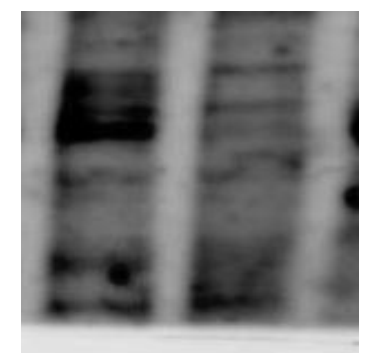
SPNS2

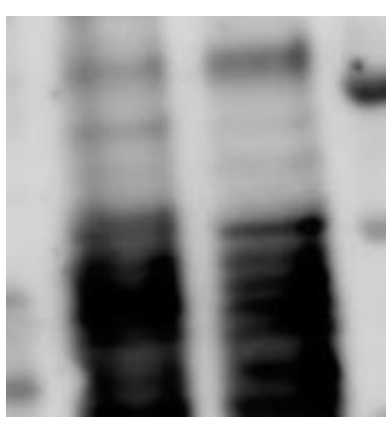
S1PR1

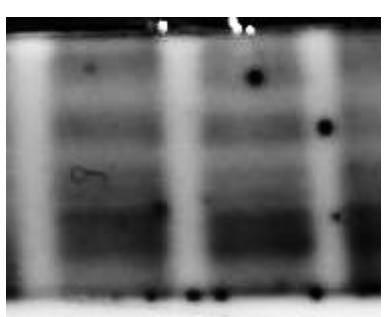
S1PR2

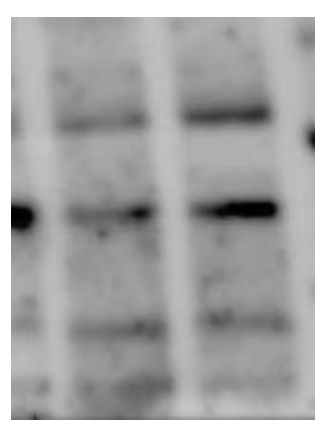
S1PR3

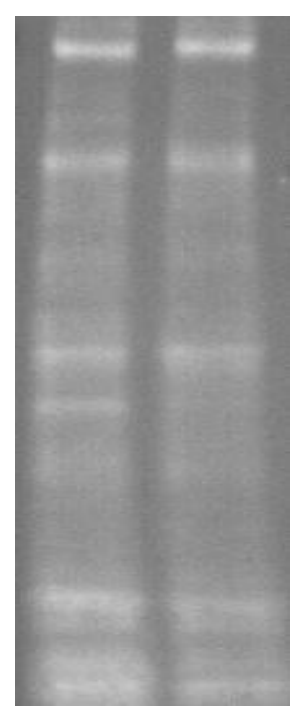
Total proteins

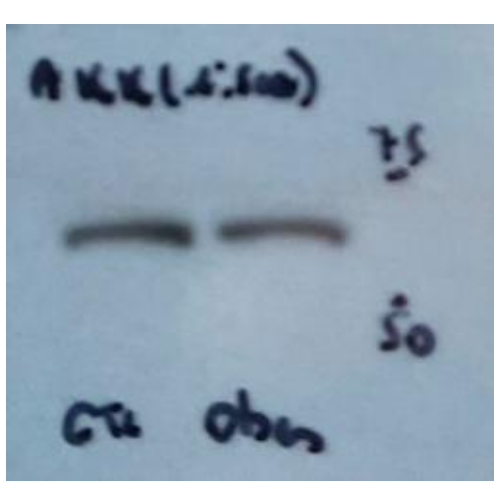
AKT1

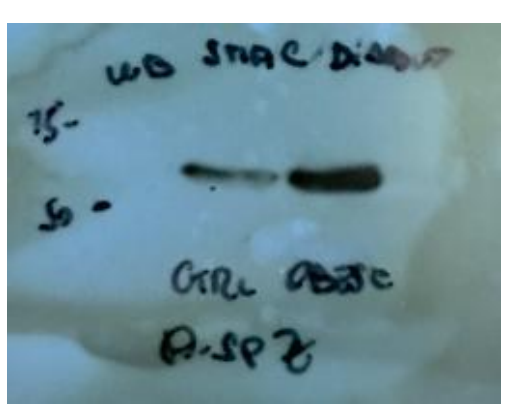
SMAC

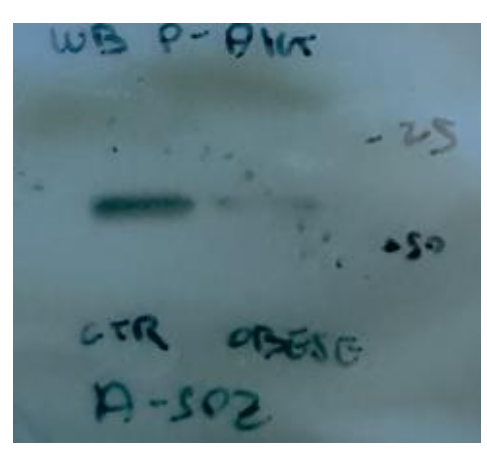
pAKT1

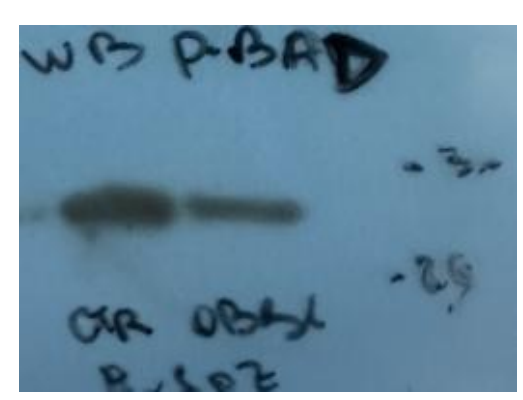
pBAD

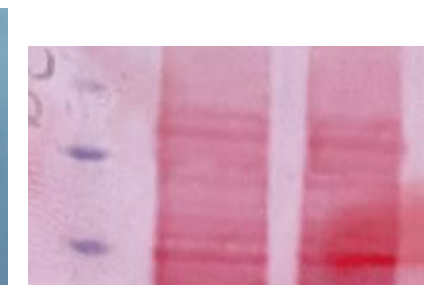
PON.S

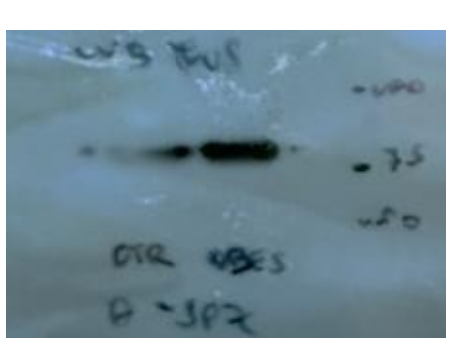
FUS

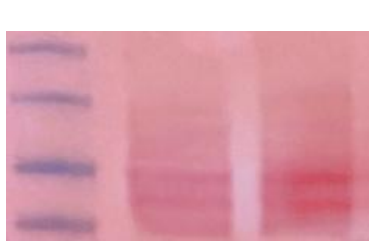
PON.S

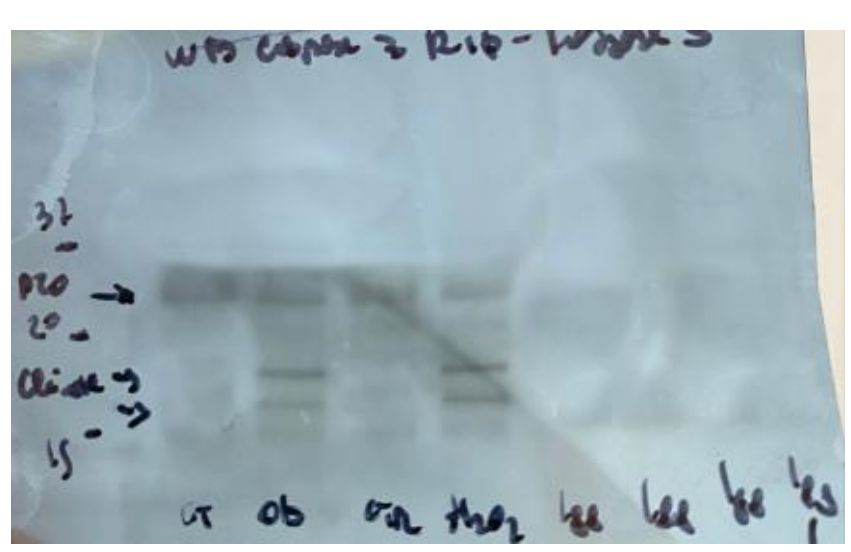
CASP3

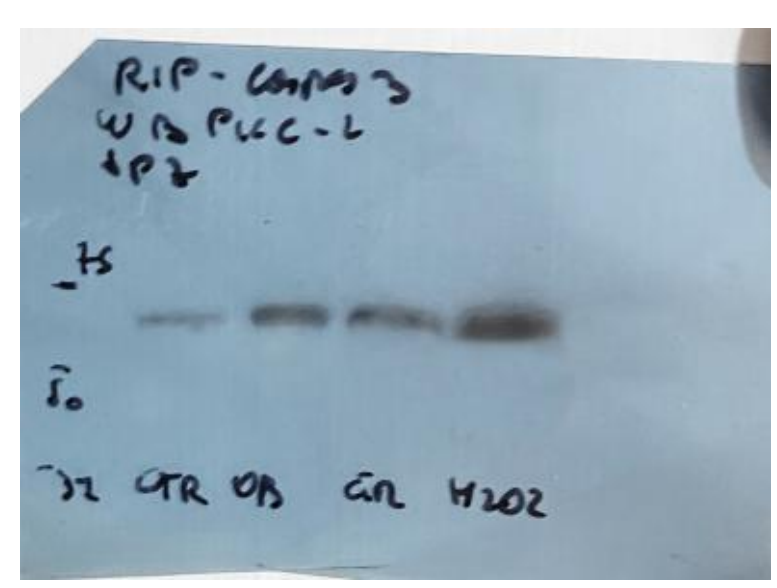
PKC
