# Supplementary material for: The sperm survival switch: circRNA-mediated regulatory loops control apoptotic signalling in human spermatozoa
Source: Cell Death Discov. 2026 Jul 25;12:362. doi: 10.1038/s41420-026-03262-0 (PMC13562583; doi:10.1038/s41420-026-03262-0)
Supplement: Supplementary file 7 [file 41420_2026_3262_MOESM1_ESM.docx]

**Supplementary Figure Legends**

**Supplemental Figure 1**. Assessment of sperm early apoptotic phenotype. (**A**) Representative immunofluorescence Annexin-V staining (green) in SPZ. (**B**) The amount of Annexin-V positive cells in Total- and A-SPZ fractions isolated by density gradient centrifugation protocol. Data were expressed as percentage and reported as mean ± SEM. ***: p<0.001

**Supplemental Figure 2**. DE-cicRNAs types and relative chromosomal distribution. (**A**) The distribution of different circRNA types among the all identified DE-circRNAs. (**B**) The distribution of up- and down-regulated DE-circRNAs in Obese A-SPZ, according to their host gene location.

**Supplemental Figure 3**. Gene ontology (GO) enrichment analysis of host genes. (**A**) The Top GO annotations in Obese A-SPZ up-regulated circRNAs. (**B**) The Top GO annotations in Obese A-SPZ down-regulated circRNAs.

**Supplemental Figure 4**. Kyoto Encyclopedia of Genes and Genomes (KEGG) pathway annotation of host genes. (**A**) The Top KEGG signalling pathway annotations in Obese A-SPZ up-regulated circRNAs. (**B**) The Top KEGG signalling pathway annotations in in Obese A-SPZ down-regulated circRNAs.

**Supplemental Figure 5**. *In vitro* experiment of vesicle shuttle: circRNA delivery from Obese EVs to Ctrl A-SPZ. Expression analysis of circCALR, circDOCK7 and circNOX4 in Ctrl A-SPZ *in vitro* treated with: i) PBS, ii) Obese seminal plasma (Obese-SP) and iii) Obese-SP pretreated with anti-CD9 antibody; qRT-PCR data were normalized using GAPDH, expressed as fold expression (nfe) and reported as mean value ± S.E.M. Experimental groups with statistically significant differences (p<0.01) were indicated with different letters.
